# Exploring the associations between preen oil bacterial, chemical and proteomic profiles of passerines

**DOI:** 10.1007/s10482-025-02182-w

**Published:** 2025-10-16

**Authors:** I. Maureen Baars, Jakub Mrázek, Jakub Kreisinger, Ivan Mikšík, Maurine W. Dietz, Joana Falcao Salles, B. Irene Tieleman, Veronika Gvoždíková Javůrková

**Affiliations:** 1 Groningen Institute for Evolutionary Life Sciences, University of Groningen, Nijenborgh 7, 9747 AG Groningen, Netherlands; 2 Institute of Animal Physiology and Genetics, Czech Academy of Sciences, Vídeňská, 1083, 160 00 Prague-Krč, Czech Republic; 3 Faculty of Science, Department of Zoology, Charles University, Viničná 7, 128 44 Prague, Czech Republic; 4 Department of Analytical Chemistry, Faculty of Chemical Technology, University of Pardubice, Studentská 573, 532 10 Pardubice, Czech Republic; 5 Institute of Vertebrate Biology of the Czech Academy of Sciences, Květná 8, 603 65 Brno, Czech Republic

**Keywords:** Preen oil microbiome, Olfactory communication, Functional proteome, Feather antimicrobial protection

## Abstract

Preen gland bacteria are thought to be the key producers of preen oil components such as chemosignalling molecules including volatile organic compounds (VOCs) and antimicrobial compounds including peptides and antimicrobial VOCs. However, data on the preen oil bacteriome and chemical composition are limited to a small subset of bird species, and the presence of antimicrobial peptides is largely unexplored. Here, we performed an exploratory study to characterize, for the first time, the preen oil chemical and proteomic profiles and to explore the possible contribution of the bacteriome to the production of preen oil VOCs and antimicrobial peptides (bacteriocins) in eight passerine species, each represented by a single individual. Preen oil bacteriome, chemical and proteomic profiles varied among birds. The bacterial profiles were dominated by the genera *Streptococcus*, *Lactococcus*, *Corynebacterium* and *Cutibacterium*. The chemical profiles mainly consisted of alcohols, ketones and carboxylic acids. The biological functions primarily associated with the proteomic profiles were proteolysis and response to oxidative stress. Although we were unable to explore a direct association between the bacteriome and chemical profiles, the preen oil contained bacteriocin- and VOC-producing bacterial genera capable of producing detected microbially-derived VOCs (mVOCs), the relative abundance of which varied between birds. Riparian species showed the highest chemical diversity and high abundances of putative preen oil mVOC-producing bacteria, which could suggest habitat-specific adaptations. This exploratory study may significantly contribute to the formulation of hypotheses on the potential role of host ecological factors in the variation of preen oil bacterial, chemical and proteomic profiles in passerines.

**Supplementary Information:**

The online version contains supplementary material available at 10.1007/s10482-025-02182-w.

## Introduction

Bacteria found in the preen glands of birds have been proposed to fulfil functional roles for the host, raising the question regarding the origin—host or bacterial—of the chemosignalling and antimicrobial functions associated with preen oil (Martín-Vivaldi et al. 2010; Whittaker et al. 2019). The preen gland, present in almost all bird species, secretes preen oil that birds spread on their plumage during preening to waterproof feathers (Jacob and Ziswiler 1982; Moreno-Rueda 2017). Besides feather maintenance and protection against mechanical wear, preen oil may also function to protect the avian host against feather degradation and infections caused by pathogenic bacteria and ectoparasites (Møller et al. 2010; Moreno-Rueda 2011, 2017; Videvall et al. 2021). There are many in vitro studies showing that preen oil inhibits the growth of plumage bacteria, including feather-degrading bacteria (Braun et al. 2018; Burger et al. 2004; Law-Brown 2001; Magallanes et al. 2016; Ruiz-Rodríguez et al. 2015; Shawkey et al. 2003; Soler et al. 2008). This antimicrobial activity may be mediated by a variety of preen oil antimicrobial compounds, including volatile organic compounds (VOCs) and proteins (Braun et al. 2018; Martín-Vivaldi et al. 2010). However, in vivo experimental studies found less consistent evidence for the antimicrobial activity of preen oil when comparing birds with and without access to their preen glands (Czirják et al. 2013; Giraudeau et al. 2013).

Preen oil might also be involved in the inter- and intraspecific communication of birds: the VOCs present in preen oil have the potential to produce odours and mediate interindividual communication (Grieves et al. 2022; Whittaker and Hagelin 2021). Recent insights have shown that birds have a functional olfactory system and that odours play a larger role in their communication than previously thought (Grieves et al. 2022; Whittaker and Hagelin 2021). For example, VOCs in preen oil have been found to serve as olfactory signals affecting mate choice, recognition, and predator and ectoparasite avoidance in birds (Grieves et al. 2022; Whittaker and Hagelin 2021).

Recent studies suggest that symbiotic bacteria in the preen gland may synthesize the chemicals, including VOCs, found in preen oil (Martín-Vivaldi et al. 2010; Whittaker et al. 2019). Bacteria that are known VOC producers were found in the preen glands of several species (Pearce et al. 2017; Rodríguez-Ruano et al. 2018; Whittaker et al. 2019), and bacteria isolated from Dark-eyed junco preen glands produced commonly found preen oil VOCs in vitro (Whittaker et al. 2019). The preen oil VOC profile and VOC abundance in preen oil were altered after the removal of the preen gland bacteriome using antibiotics in Eurasian hoopoes and Dark-eyed juncos, respectively (Martín-Vivaldi et al. 2010; Whittaker et al. 2019). This suggests that preen gland bacteria might be responsible for the production of at least part of the preen oil VOCs.

In addition to VOCs, the preen gland bacteria may produce other antimicrobials such as bacteriocins (ribosomally synthesized antimicrobial peptides) (Martín-Vivaldi et al. 2010; Soler et al. 2008). Bacterial strains isolated from preen glands of multiple bird species showed antimicrobial activity against indicator bacterial strains (including feather degrading bacteria) in vitro (Bodawatta et al. 2020; Martínez-Renau et al. 2022; Ruiz-Rodríguez et al. 2013, 2012; Soler et al. 2024), and in some strains this antimicrobial activity is mediated by the production of bacteriocins (Martín-Platero et al. 2006; Ruiz-Rodríguez et al. 2013, 2009).

The bacteriome of the preen gland thus likely produces VOCs and other antimicrobial compounds that help birds prevent feather degradation (VOCs and other antimicrobial compounds) and play a role in inter- and intraspecific communication (VOCs). Yet, detailed knowledge of the bacterial communities in the preen gland is limited to only a few bird species (Bodawatta et al. 2020; Grieves and Gloor^2025^; Grieves et al. 2021; Pearce et al. 2017; Rodríguez-Ruano et al. 2018; Videvall et al. 2021; Whittaker et al. 2019). Additionally, data on the presence of antimicrobial peptides in preen oil are limited to only two peptides in one bird species (House sparrow) (Carneiro et al. 2020), and no data exist on the complete proteomic profile of preen oil. Consequently, we have no insight into the variation in preen gland bacteriome composition, chemical (including VOCs) and proteomic profiles between birds, and the potential causes for this variation. Exploring preen oil bacterial, chemical and proteomic profiles in different bird species will be the foundation for the postulation of hypotheses on the causes of variation in preen gland bacteriome composition and preen oil chemicals (including VOCs) and antimicrobials in birds.

In this pilot study, we aim to i) explore the composition of the preen oil bacteriome, chemicals (including VOCs) and proteomic profiles, and ii) explore the association between the preen oil bacteriome and the presence of microbially derived preen oil VOCs (mVOCs) and antimicrobial peptides (represented by bacteriocins) in eight species of passerines.

## Materials and methods

### Study species and data collection

In total, we sampled 20 individuals (17 males:3 females) of 8 passerine species at 8 sites in the Czech Republic during March–May 2015–2016 (Table S1; Electronic Supplementary Material). The numbers of individuals and species used for preen oil analyses were as follows: 8 individuals of 8 species for the bacteriome profiling, 7 individuals of 7 species for the chemical/VOC profiling and 5 individuals of 4 species for the proteomic profiling (see Electronic Supplementary Table S1 for details). Since we have one individual per species for each type of analysis, we refer to variation between samples as variation between birds, as we cannot distinguish between inter- and intra-specific variation in our study. We captured all individuals using mist nests during their pre-breeding period (1–3 weeks prior to the typical peak breeding dates in the study region (Šta̕stný and Hudec 2011); see Electronic Supplementary Table S1) to minimise the confounding effects of seasonal differences in the chemical profile of preen oil (Potier et al. 2018; Soini et al. 2007; Tuttle et al. 2014). We removed each bird from the mist net, immediately placed it into a clean cloth bag and collected preen oil using a sterile 10 μl end-to-end glass capillary tube (Hirschmann Laborgeräte, Germany) wearing sterile nitrile gloves. We cut off the end of each capillary containing preen oil, placed it in a dry, sterile 1.8 ml screw-capped cryotube (Thermo Scientific – Nunc) with a rubber O-ring sealing to minimize the loss of VOCs, and stored it in a cooler at 5 °C for < 6 h. We then stored the cryotubes at − 20 °C until the preen oil bacterial, volatile and proteomic profiling. Although all sampling and storage procedures were carried out with maximal caution, it is worth noting that losses of some very volatile organic compounds (VVOCs) occurred. This may result in incomplete VOC profiles presented in this study, as these compounds may be missing. However, it is impossible to fully prevent this during the sampling of preen oil from free-living birds.

### Preen oil bacteriome analysis

We extracted DNA from preen oil using the RTP® Bacteria DNA Mini kit (STRATEC Molecular GmbH, Berlin, Germany) following the isolation kit’s Protocol 5 (Isolation of microbial DNA from tissue biopsies). Negative DNA extraction was done along with the samples. For each sample (including the negative control), we diluted the isolated DNA tenfold in nuclease-free H_2_O (Qiagen, Hilden, Germany), from which we used 2 µL as a template in the PCR reaction. Next, we amplified the bacterial variable V4–V5 region of 16S rRNA gene using the specific primer pair BactB-F (GGATTAGATACCCTGGTAGT) and BactB-R (CACGACACGAGCTGACG) (Fliegerova et al. 2014), and performed the PCR reaction using EliZymeTM HS FAST MIX Red Master Mix (Elisabeth Pharmacon, Brno, Czech Republic). Thermal cycling conditions included an initial denaturation for 5 min at 95 °C, followed by 25 cycles consisting of 30 s at 95 °C, 30 s at 57 °C, 30 s at 72 °C, and a final elongation step at 72 °C for 5 min. We checked the length and quality of the amplicons by agarose gel electrophoresis (1.5%) and purified the PCR products using the Monarch® PCR & DNA Cleanup Kit (New England BioLabs, Ipswich, MA, USA). The negative DNA control did not amplify successfully and was therefore excluded from the library preparation.

We performed the library preparation using the NEBNext Fast DNA Library Prep Set for Ion Torrent (New England BioLabs, Ipswich, MA, USA) and the Ion Xpress Barcode Adapters 1–96 Kit (Thermo Fisher Scientific, Waltham, MA, USA). We analysed the length of the target amplicons of DNA libraries using the 2100 Bioanalyzer Instrument (Agilent Technologies, Santa Clara, CA, USA) and pooled the amplicons in equimolar ratios based on concentration determined using a KAPA Library Quantification Kit (KAPA Biosystems, Roche, Pleasanton, CA, USA). The template amplification and enrichment were performed by emulsion PCR in the Ion OneTouch™ 2 instrument using the Ion PGMTM HiQTM View OT2 Kit-400 (Thermo Fisher Scientific, Waltham, MA, USA). The enriched template was sequenced with the Personal Genome Machine (PGM™) System (Thermo Fisher Scientific,Waltham, MA, USA) using the Ion PGM™ Hi-Q™ View Sequencing solutions kit and the Ion 316™ Chip v2 BC according to the manufacturer’s protocols.

We first detected and trimmed gene-specific primer sequences with a skewer (Jiang et al. 2014). Using the R package *dada2* (Callahan et al. 2016), we trimmed all reads to the same length (260 bp), eliminated low-quality reads (expected error rate per paired-end read > 2), and denoised quality-filtered reads using the parameters recommended for Ion Torrent data by the dada2 developers (i.e., HOMOPOLYMER_GAP_PENALTY = − 1 and BAND_SIZE = 32). We then created an abundance matrix that included the number of reads for each 16S rRNA amplicon sequencing variant (ASVs) in each sample. Using Uchime (Edgar et al. 2011) in conjunction with the gold.fna reference database (available at: https://drive5.com/uchime/gold.fa), we detected and eliminated chimeric ASVs. Subsequently, we assigned the taxonomy for non-chimeric ASVs with 50% posterior confidence by the RDP classifier (Wang et al. 2007) using the Silva database v.138 (Quast et al. 2012) as a reference.

We performed further filtering in QIIME2 (v 2022.11) (Bolyen et al. 2019), where we filtered ASVs assigned to Archaea, Eukaryota, Mitochondria, and Chloroplasts, as well as the ASV assigned to *Delftia* sp., as this abundant ASV, present in all samples, is often found to be a contaminant from DNA extraction kits (Salter et al. 2014). Next, we rarefied the ASV table at a sequencing depth of 2765 reads, based on alpha rarefaction curves (Fig. S1; Electronic Supplementary Material) that saturated at 2000 reads for both the number of observed ASVs and Shannon diversity and the read count of the first sample with read counts > 2000 (2769 reads). Rarefying removed 1 sample from the dataset (*Acrocephalus arundinaceus*), leaving 7 samples from 7 passerine species*.*

### Preen oil untargeted chemical/VOC profiling

Preen oil samples were processed in a service laboratory (Mass Spectrometry Laboratory of the University of Chemistry and Technology Prague) using stir bar sorptive extraction (SBSE) coupled with gas chromatography-mass spectrometry (GC–MS), with slightly modified conditions, described in Soini et al. 2013 (Soini et al. 2013). Briefly, the glass microcapillary tube containing the preen oil sample was placed into a 20 mL capped glass vial with 2 mL of high-purity water and a Twister™ stir bar (10 mm, 0.5 mm film thickness, 24 μL polydimethylsiloxane, PDMS, volume). Adding 100 mg of ammonium sulphate was omitted for most of the samples because a pre-experimental trial with one preen oil sample originating from Sand Martin uncovered a problem with this method. Despite rinsing the stir bar with distilled water, ammonium sulphate remained on the surface of the stir bar. During subsequent thermal desorption, ammonium sulphate decomposed into NH_3_ and SO_2_, and especially the presence of NH_3_ caused significant “bleeding” of the CG column (mostly visible on one sand martin chromatogram), leading to rapid degradation of the stationary phase of the GC column and Twister™ stir bar destruction. As an internal standard, 8 ng of 7-tridecanone (Sigma Aldrich, Germany) was added to 5 μL of methanol in each vial. The sample was stirred at 800 rpm on an HSC 7 VELP magnetic stirrer (VELP Scientifica, Italy) for 60 min. Before extraction, all glassware was washed with acetone and dried at 80 °C. After extraction, the stir bars were gently rinsed with a small amount of distilled water, dried on paper tissue and placed in the Thermal Desorption Autosampler tubes (Gerstel GmbH, Germany) for subsequent gas-chromatography–mass spectrometric (GC–MS) analysis. Together with the samples, a standard mixture of n-alkanes (49,452-U Supelco, Sigma Aldrich, Germany) was analysed under the identical GC–MS conditions specified below to determine the retention behaviour of the individual analytes contained in the preen oil samples on the GC column.

For GC–MS profiling, a Trace GC Ultra (Thermo Scientific) gas chromatograph connected to an ISQ mass spectrometer (Thermo Scientific) with a Thermal Desorption Autosampler with a Cooled Injection System (TDSA–CIS 4) was used. The separation capillary was DB-5MS Agilent (30 m × 0.25 mm, i.d., 0.25 m film thickness). Samples were thermally desorbed in a TDSA automated system, followed by a splitless injection into the column with a CIS-4 cooled injection assembly. The temperature separation program on the GC column was as follows: 50 °C (4 min), then increasing to 290 °C at the rate of 10 °C/min 3 °C/min (hold time: 30 min).

To identify the organic compounds in the preen oil samples, the measured mass spectra of individual chromatographic peaks (controlled also against n-alkanes standard RIs) were compared with the NIST 14 mass spectra database (https://chemdata.nist.gov/) with match factor cut-off scores ranging between 900 and 950 (i.e. excellent match). Blank measurements of the Twister and all backgrounds were subtracted from the chromatograms before further processing. We assigned our identified organic compounds as (semi-)volatile organic compounds ((S)VOC) if the compound vapour pressure at a temperature of 293.15 K (20 °C) is between 10^–8^ and 10^–2^ kPa (sVOC) or more than 10^–2^ kPa (VOC) (Directive 2010/75/EU; Liu 2022). All identified compounds were assigned as (S)VOCs, except for 3 compounds for which no data on vapour pressure at 293.15 K was available (Table S3; Electronic Supplementary Material). The identification of compounds based on comparing measured mass spectra with the NIST 14 mass spectra database was only possible for compounds with retention times (RTs) up to 25 min (Fig. S2; Electronic Supplementary material). The organic compounds with RTs > 25 min can only be identified as a heterogeneous mixture of fatty acid alkyl esters. For accurate identification of these compounds, it is necessary to measure the standards under the same chromatographic conditions and then compare the retention times and mass spectra of these standards with the measured data from the preen oil analyses. Since our study focused only on the identification of preen oil (S)VOCs, and since the compounds with a retention time (RT) > 25 min (of non-volatile nature) could not be identified with certainty, the estimation of relative abundances of detected (S)VOCs in the complete profiles would be highly biased. Therefore, we limited our analysis to the presence/absence data of the (S)VOCs detected in the preen oil of the species studied.

### Preen oil proteomic profiling

We performed preen oil proteomic profiling using LC–MS/MS. Specifically, the nano-HPLC apparatus used for protein digest analysis was a Proxeon Easy-nLC (Proxeon, Odense, Denmark) coupled to an ultrahigh resolution MaXis Q-TOF (quadrupole—time of flight) mass spectrometer (Bruker Daltonics, Bremen, Germany) by nanoelectrosprayer. We used the software packages HyStar 3.2 and micrOTOF-control 3.0 to control the nLC-MS/MS instruments and collected and manipulated the data with the software packages ProteinScape 3.0 and DataAnalysis 4.0 (Bruker Daltonics). We used the following chemicals during the analysis; trypsin (TPCK treated, from bovine pancreas, 13,500 units per mg), ammonium bicarbonate and acetonitrile (HPLC–MS grade) obtained from Sigma (St. Louis, MO, USA) and prepared all solutions in MilliQ water (Millipore, Bedford, MA, USA).

We inserted the capillaries with preen oil samples into glass vials with 30 μl of trypsin buffer (0.2 mg/ ml trypsin in 100 mmol/L ammonium bicarbonate pH 7.8), washed the contents of the capillaries out five times with this buffer (centrifugated at 6000 g) and treated the contents with trypsin buffer for 18 h at 37 °C. After trypsin cleavage, we extracted peptides from the samples with StageTips using Empore C18 Extraction disks (Supelco; Bellefonte, PA, USA) according to the published protocol (Rappsilber et al. 2007).

We injected five microliters of the peptide mixture into an NS-AC-12dp3-C18 Biosphere C18 column (particle size: 3 µm, pore size: 12 nm, length: 200 mm, inner diameter: 75 µm) with an NS-MP-10 Biosphere C18 precolumn (trap-column) (particle size: 5 µm, pore size: 12 nm, length: 20 mm, inner diameter: 100 µm), both manufactured by NanoSeparations (Nieuwkoop, The Netherlands). The separation of peptides was achieved via a linear gradient between mobile phase A (water) and B (acetonitrile), both containing 0.1% (v/v) formic acid. We started separation by running the system with 5% mobile phase B, followed by a gradient elution to 7% B at 5 min and 30% B at 180 min. The next step was a gradient elution to 50% B in 10 min and then a gradient to 100% B in 10 min. Finally, we eluted the column with 100% B for 20 min. We achieved equilibration between the runs by washing the column with 5% mobile phase B for 10 min. The column was held at ambient temperature (25 °C), and the flow rate was 0.20 µL/min. We used online nano-electrospray ionization (easy nano-ESI) in positive mode. We set the ESI voltage to + 4.5 kV, the scan time to 3 Hz, the drying gas (N_2_) to 4 L/min, the drying gas temperature to 180 °C, and the nebulizer pressure to 100 kPa. Measurements were performed by scanning from 50 to 2200 m/z. The reference ion (internal mass lock) was a monocharged ion of C_24_H_19_F_36_N_3_O_6_P_3_ (m/z 1221.9906). We averaged mass spectra corresponding to each signal from the total ion current chromatogram for an accurate molecular mass determination. We conducted all LC–MS and LC–MS/MS analyses in duplicate.

We processed the data using ProteinScape software v. 3.0.0.446 (Bruker Daltonics, Bremen, Germany). We identified proteins by correlating tandem mass spectra to the extracted database for *Aves* from the NCBI database (downloaded on 30th January 2017—2,198,542 sequences; 1,077,083,019 residues), using the MASCOT searching engine v. 2.3.0 (http://www.matrixscience.com). We used the whole UniProt database for control searching, choosing trypsin as the enzyme parameter, allowing three missed cleavages, and using an initial peptide mass tolerance of ± 10.0 ppm for MS and ± 0.05 Da for MS/MS analysis. We set the following modifications to be variable: proline and lysine were allowed to be hydroxylated and methionine oxidated, whereas asparagine and glutamine were deamidated. We set the monoisotopic peptide charge to 1 + , 2 + and 3 + , and selected the Peptide Decoy option during the data search process to remove false-positive results. Only significant hits were accepted (MASCOT score ≥ 80 for proteins and MASCOT score ≥ 20 for peptides, http://www.matrixscience.com). All identified proteins and their sequences were manually validated using the peptide search tool of the UniProt database (release 2023_03). We listed identified proteins with the highest UniProt BLAST similarities, along with accompanying information (i.e., primary NCBI, UniProtKB and UniParc accession numbers, source organism, evidence for protein existence, annotation score and its status (unreviewed—TrEMBL or reviewed-UniProtKB/Swiss-Prot) in summarizing tables (Table S2, Electronic Supplementary Material).

### Statistics and data analysis

We calculated Jaccard distances with rarefied data using the QIIME2 pipeline. The QIIME2 output was imported into R statistical software v 4.2.2 (*qiime2R* R package v 0.99.6) for further analysis and visualisation. We generated taxa barplots of the relative abundance at the phylum, class and genus level, as well as a heatmap of the relative abundance at the genus level, using the non-rarefied feature table and taxonomy assignment (*ggplot2* R package v 3.4.1 (Wickham 2016)). We used the rarefied feature table to create a shared ASV Upset plot (*ComplexUpset* R package v 1.3.3 (Krassowski 2021)).

To assess the potential functional characteristics of the preen oil proteomic profiles, we used the complete list of UniprotKB and UniParc accession numbers for all proteins identified in the preen oil of specific passerine species as a source to visualise the Gene Ontology (GO) of the identified preen oil proteins using the *UniprotR* R package (Soudy et al. 2020).

We combined the preen oil bacteriome data with chemical profiling data to determine whether the bacterial genera found in preen oil have the potential to produce the VOCs detected in preen oil. First, we entered all compounds found in preen oil into the microbial VOC (mVOC) 4.0 Database (Kemmler et al. 2025) and recorded which bacterial genera were found to produce these VOCs. Then, based on data from the mVOC database, for each bacterial genus detected in preen oil from a particular species, we determined whether that bacterial genus could produce VOCs found in preen oil. With this data, we created pie charts (*ggplot2* v 3.4.1 (Wickham 2016)) of the relative abundance of the bacterial genera found in each bird species, including the information on whether the bacterial genus has the potential to produce preen oil mVOCs. To further assess the relationship between preen oil bacterial communities and VOC profiles, we assessed differences in chemical profiles between species using the Jaccard dissimilarity index based on presence/absence data (*vegan* R package v 2.6–4 (Oksanen et al. 2022)). We then performed a Procrustean analysis on the PCoA of chemical data and the PCoA of bacteriome data (Jaccard). To determine the significance of the association between preen oil bacterial communities and chemical profiles, we used a permutation procedure for Procrustean rotation (PROTEST) with 999 permutations (*vegan* v 2.6-4 (Oksanen et al. 2022)) (Jackson 1995; Peres-Neto and Jackson 2001). We created shared chemical compound Upset plots using the *ComplexUpset* package v 1.3.3 (Krassowski 2021).

To determine the potential of the preen oil bacteriome to produce bacteriocins, we searched all bacterial genera found in preen oil in the BACTIBASE database (Hammami et al. 2010). Each genus that was found to produce at least one bacteriocin, was indicated as putative bacteriocin producing. Next, we created pie charts with relative abundances of bacteriocin-producing bacterial genera (R package *ggplot2* R package v 3.4.1 (Wickham 2016)).

## Results

### Preen oil bacteriome

**Fig. 1:**
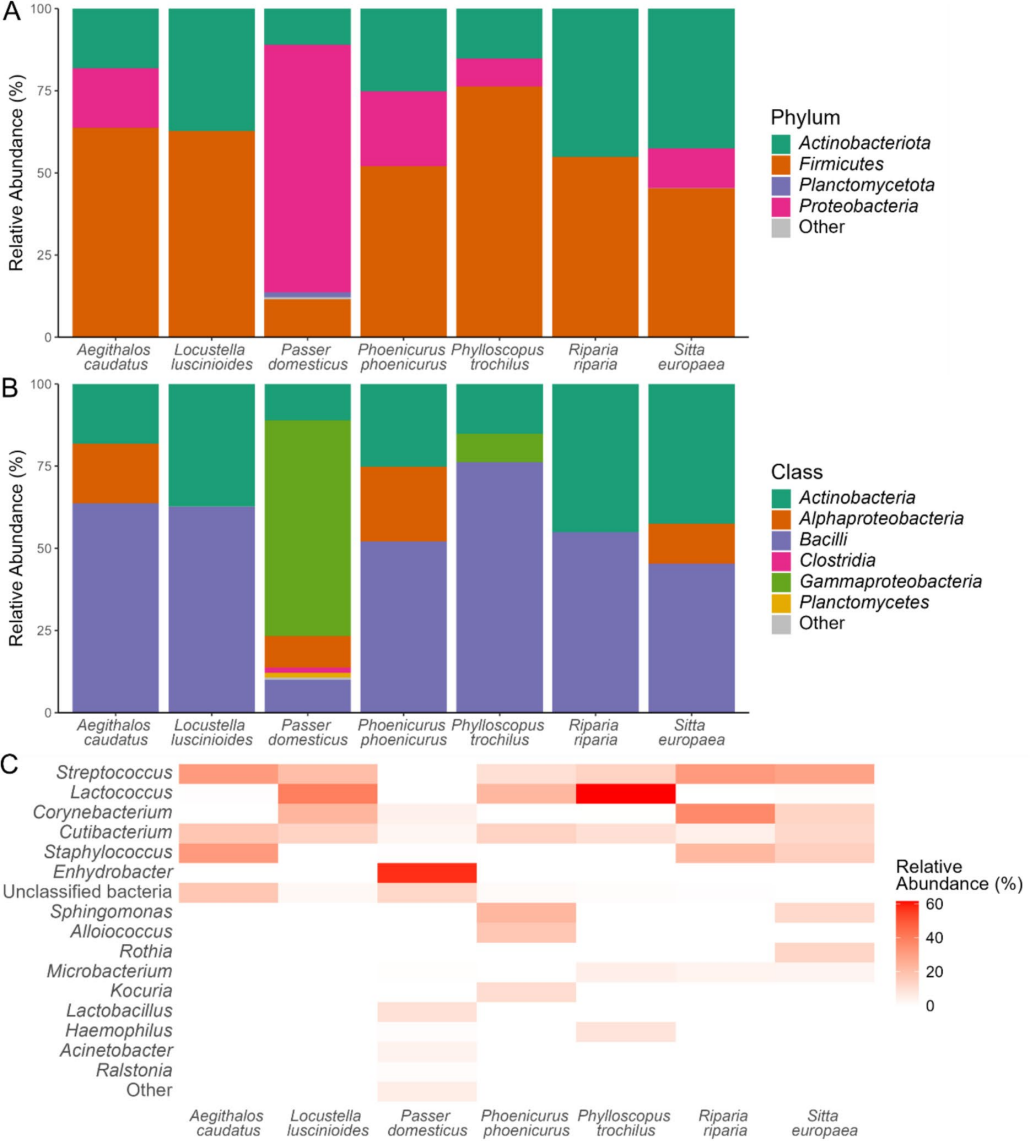
Preen oil bacteriome community composition in different passerine species expressed as variability in relative abundances of amplicon sequence variants (ASVs) (%) of **A** Bacterial phyla, **B** Bacterial classes, and **C** Bacterial genera

*Firmicutes* (11–76%) and *Actinobacteriota* (11–45%) were the most abundant phyla in the preen oil bacteriome composition in most of the passerine species (Fig. 1A). *Proteobacteria* were found in preen glands of 5 of the 7 species, and were not detected in the Savi’s warbler (*Locustella luscinioides*) and Sand martin (*Riparia riparia*). The preen oil bacteriome of the House sparrow had a relatively high abundance of the phylum *Proteobacteria* (75%). The composition of the House sparrow’s bacteriome was also unique at the class and genus level (Fig. 1B, C). For example, the genus *Enhydrobacter* (phylum *Proteobacteria*) was exclusively found in the House sparrow. On the other hand, *Streptococcus* (phylum *Firmicutes*) was found in all species except the House sparrow. *Lactococcus* (phylum *Firmicutes*) was highly abundant in the Willow warbler (*Phylloscopus trochilus*) (Fig. 1C).

**Fig. 2:**
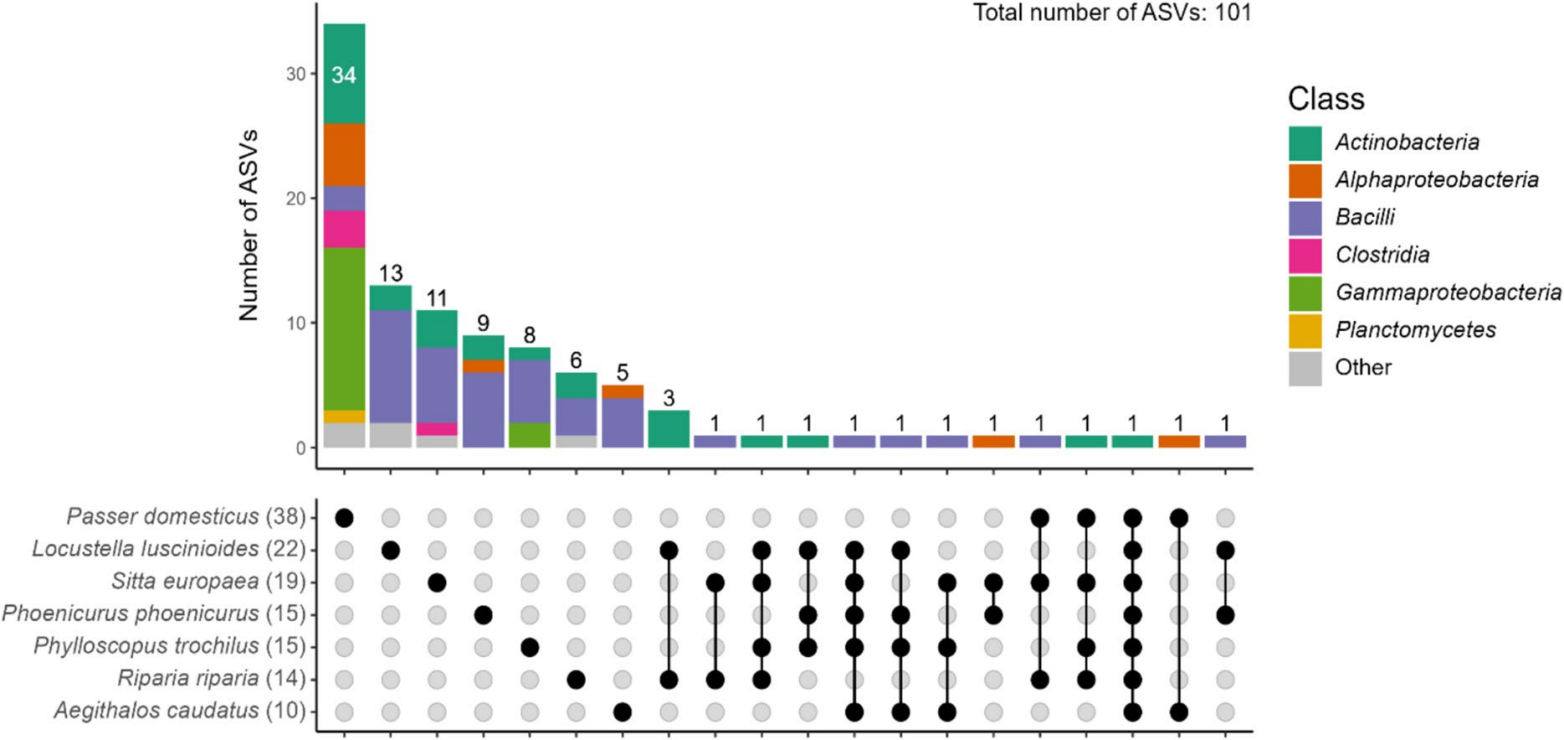
Upset plot showing the number of shared and unique amplicon sequence variants (ASVs) in the preen oil bacteriome of studied passerine species, where each ASV is coloured by bacterial class. The total number of ASVs per individual is indicated in brackets following the species name

Only one ASV (ASV28, *Genus*: *Cutibacterium*) was shared between all species (Fig. 2), and generally, most ASVs were unique to individuals. The House sparrow had the highest number of unique ASVs compared to the other birds.

### Preen oil chemical/VOC profiles

**Fig. 3:**
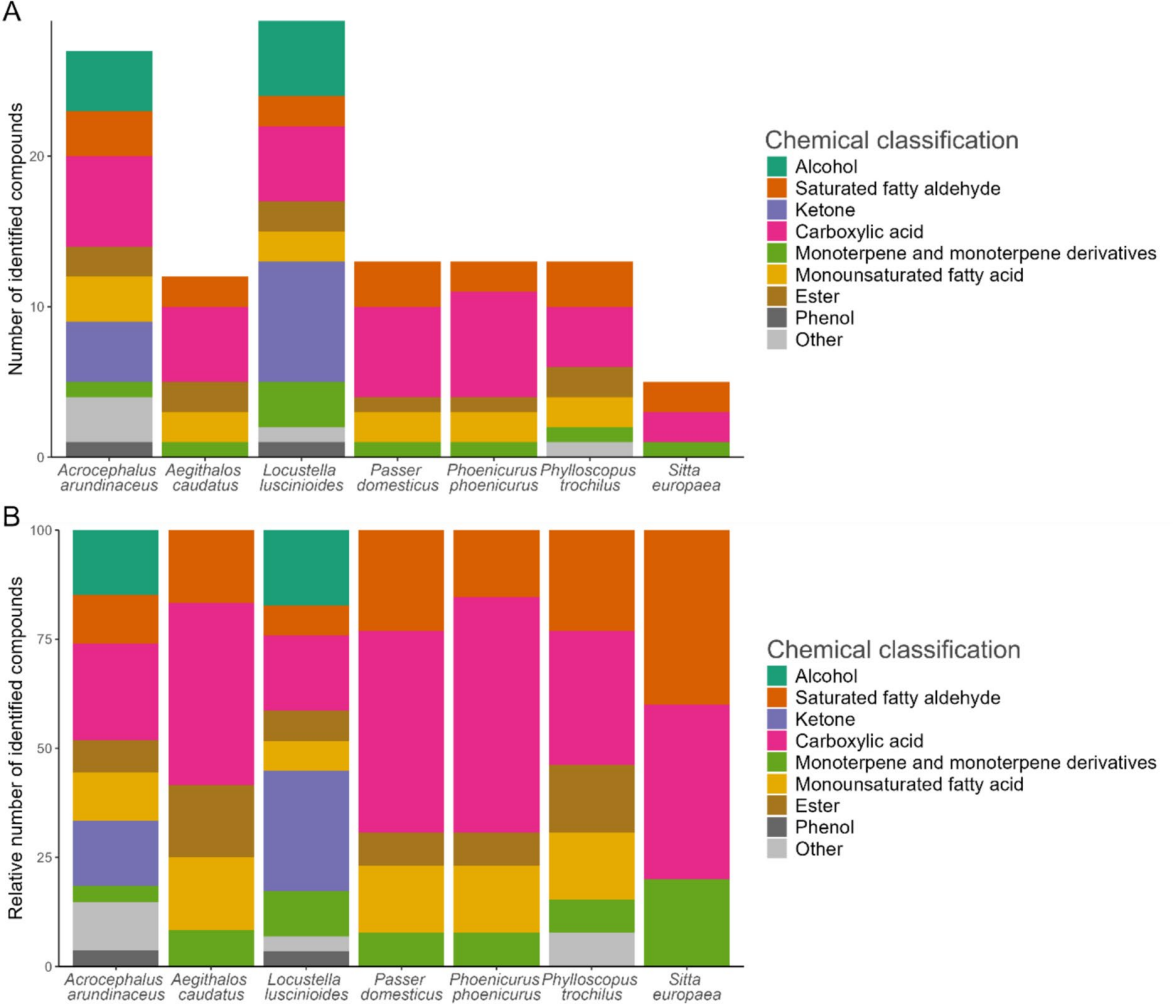
Barplots of (**A**) absolute and (**B**) relative numbers of identified organic compounds that were mostly identified as (S)VOCs, coloured by their higher chemical classification, in preen oil of studied passerine species

The number of distinct, identified compounds found in each species ranged from 29 in the Savi’s warbler to 5 in the European nuthatch (*Sitta europaea*) (Fig. 3, Fig. S3 and Table S3; Electronic Supplementary Material). In total, 38 distinct compounds were found in the preen oil of 7 species (Fig. 3, Fig. S3 and Table S3; Electronic Supplementary Material). All preen oil compounds for which data on vapour pressure is available (89% of compounds) were identified as (S)VOCs, and 73% of all preen oil compounds were found in the mVOC 4.0 database indicating that most of the identified preen oil compounds have a high degree of volatility. The major compound classes detected were alcohols, aldehydes, ketones, and carboxylic acids; the alcohols and ketones were exclusively found in the Great reed warbler and Savi’s warbler (Fig. 3, Fig. S3 and Table S3; Electronic Supplementary Material).

**Fig. 4:**
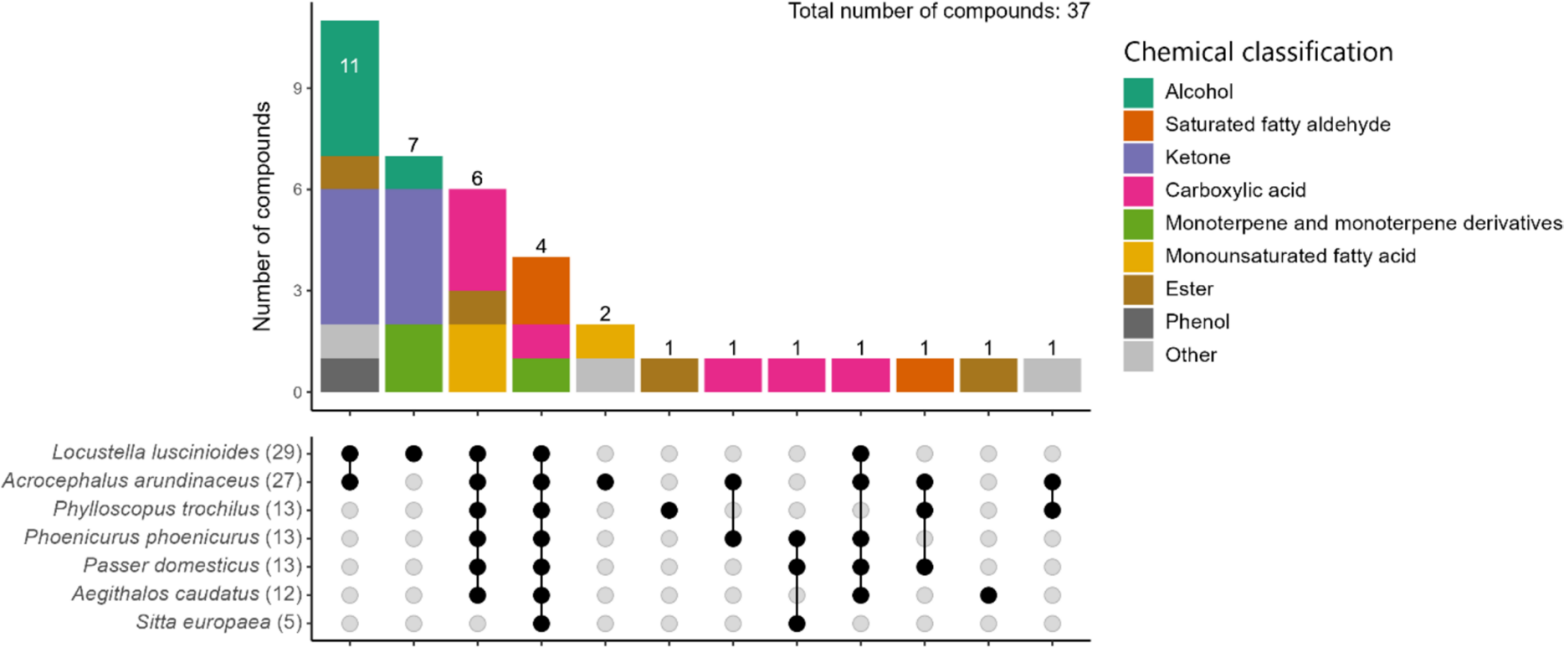
Upset plot showing the number of distinct shared and unique chemical compounds in preen oil of studied passerine species, where each compound is coloured by their higher chemical classification. The total number of compounds per individual is indicated in brackets following the species name

Four compounds were shared between all 7 birds (Fig. 4). The Savi’s warbler and Great reed warbler shared 11 compounds, and the Savi’s warbler had the highest number of compounds unique to that individual (7). The difference in preen oil VOC profiles between birds is also apparent from the chromatograms (Fig. S2; Electronic Supplementary Material).

### Preen oil proteomic profiles

The Long-tailed tit (*Aegithalos caudatus*) and Willow warbler showed the highest number of proteins identified (41 and 30 respectively, see Table S2; Electronic Supplementary Material), while the lowest number of proteins were identified for the Sand martin individuals (14 and 16; Table S2; Electronic Supplementary Material). None of the proteins identified in the preen oils were related to proteins produced by microorganisms. Conversely, all preen oil proteins had the highest similarity to those derived from birds (Table S2; Electronic Supplementary Material).

**Fig. 5:**
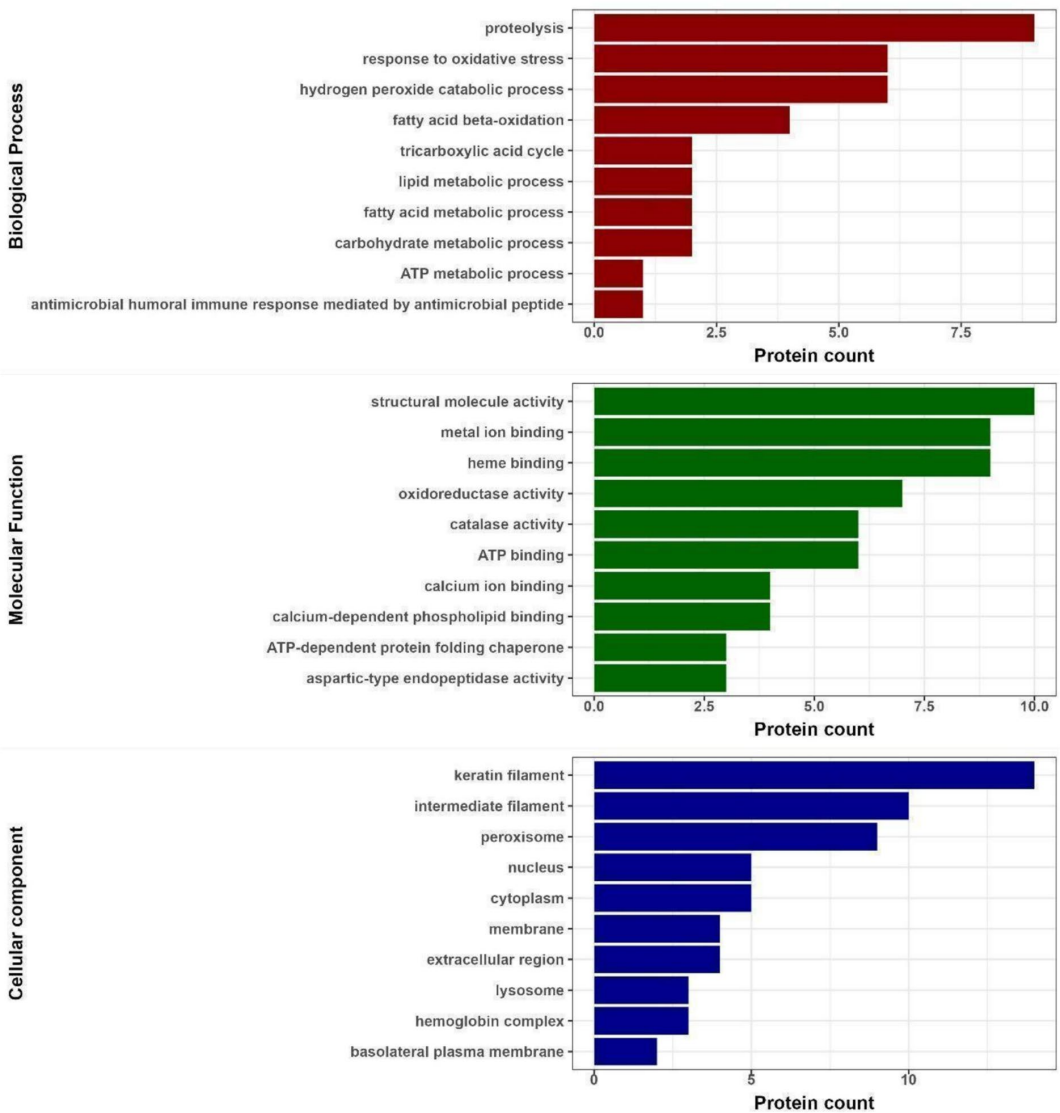
Gene ontology (GO) inferred functional properties (red = biological processes, green = molecular functions, blue = cellular component) of proteins detected in preen oil of four studied passerine species

Lysozyme was the only antimicrobial protein that we detected in preen oil, and it was only present in the Long-tailed tit (Table S2; Electronic Supplementary Material). Biological processes of all proteins identified in passerine preen oil inferred from GO analysis differed among birds (Fig. S4; Electronic Supplementary Material) and were dominated with proteolysis, response to oxidative stress, hydrogen peroxide catabolic process and fatty acid beta-oxidation (Fig. 5). Most of the preen oil proteins were identified as cellular components of keratin filament, intermediate filament and peroxisome and their molecular function was dominated by structural molecular activity, metal ions and heme binding followed by oxidoreductase and catalase activity (Fig. 5).

### Preen oil bacteriome and associated preen oil mVOCs and bacteriocin production

**Fig. 6:**
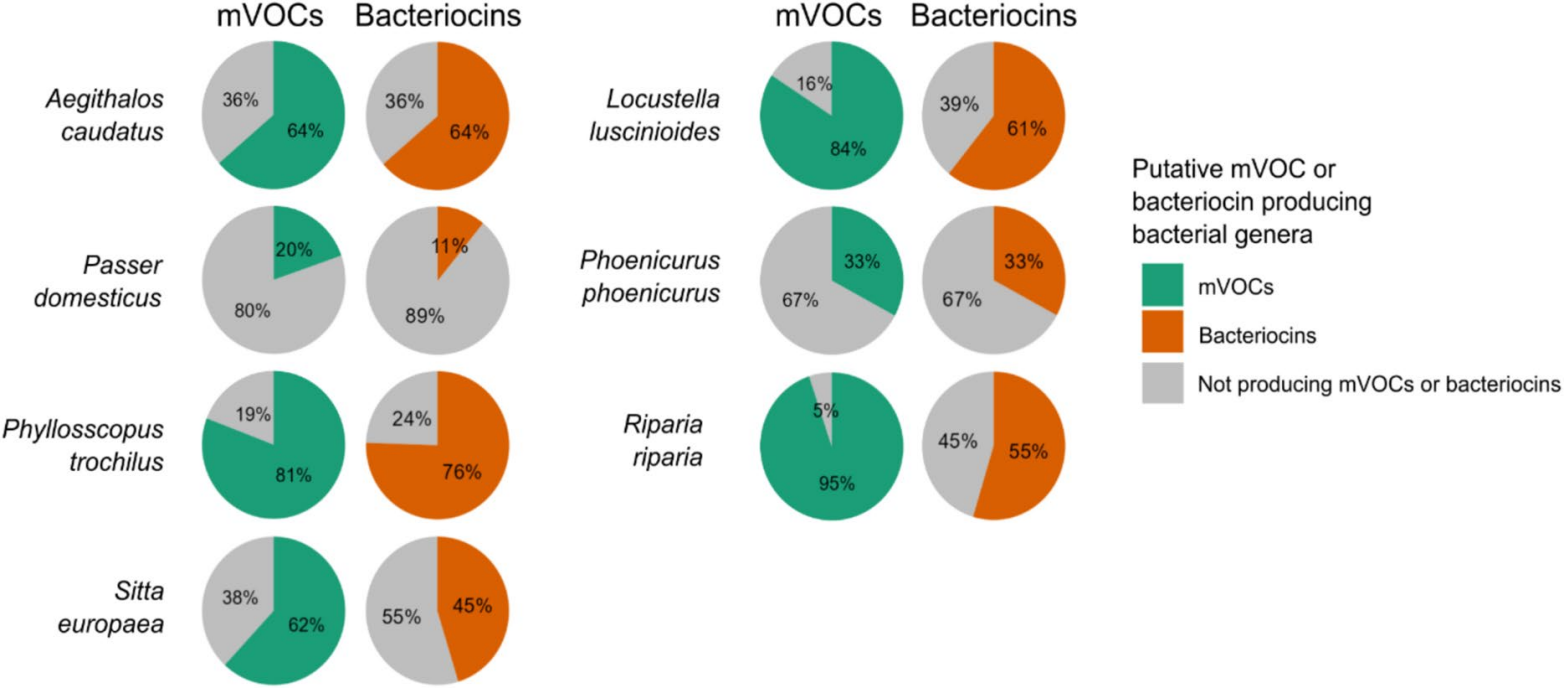
Relative abundances of putative preen oil bacteriocin-producing bacterial genera (orange) as inferred from the BACTIBASE database and microbial VOC-producing bacterial genera (green) capable of producing preen oil mVOCs as inferred from the mVOC database and chemical profiles of studied passerines. Grey indicates the relative abundance of bacterial genera with no potential to produce bacteriocins or mVOCs

The Sand martin and Savi’s warbler bacteriome had the highest potential for producing preen oil mVOCs with 95% and 84% relative abundance of putative mVOC-producing genera, respectively. On the other hand, in the House sparrow bacteriome only 20% of the genera present had the potential to produce preen oil mVOCs (Fig. 6). The Procrustes analysis did not find a significant concordance between preen oil bacteriome community structure and preen oil chemical profile (*protest*: m_12_ = 0.73, *P* = 0.53), indicating that there may be no or a weak association between preen oil bacteriome composition and preen oil chemical profiles. However, considering the lack of replicates and the fact that the bacteriome and chemical profiles were analysed from different individuals, these results should be taken with caution (Fig. S5; Electronic Supplementary Material).

The House sparrow had the lowest relative abundance of putative bacteriocin-producing bacteria (11%), while in the preen oil of the Willow warbler, 76% of the bacterial genera are putative bacteriocin producers. (Fig. 6).

## Discussion

Our exploratory study revealed substantial variation in the preen oil bacteriome of passerine birds, where *Firmicutes* and *Actinobacteriota* were the most dominant phyla. Likewise, the preen oil chemical compounds varied in profile and diversity among birds, just as the proteomic profile. We identified a total of 39 distinct chemical compounds (mostly assigned as (S)VOCs) consisting of alcohols, aldehydes, ketones, and carboxylic acids, as well as 108 unique proteins primarily associated with proteolysis, response to oxidative stress, hydrogen peroxide catabolic process and fatty acid beta-oxidation. Interestingly, we found that the preen gland bacteriome can potentially produce VOCs and bacteriocins found in passerine preen oil. To further understand how much of the variation in these traits can be attributed to species and individuals, or to ecological factors and life-history traits of birds, requires follow up studies.

### Variability of preen oil bacteriome

Here, the preen oil bacteriome varied among 8 passerine birds from 8 species (one individual per species), raising the question how much of this variation is attributable to the levels of species and individual (Bodawatta et al. 2020; Videvall et al. 2021). There is strong evidence for between-species variability in bacteriomes of preen glands, feathers and skin, in both free-living and captive birds (Engel et al. 2018; Grieves and Gloor 2025; Javůrková et al. 2019; Roggenbuck et al. 2014), but see (van Veelen et al. 2017). The composition of the preen oil bacteriome at the phylum level is consistent with those found in other studies, with *Firmicutes* and *Actinobacteriota* being the most dominant phyla (Bodawatta et al. 2020; Grieves et al. 2021; Pearce et al. 2017; Rodríguez-Ruano et al. 2018; Videvall et al. 2021; Whittaker et al. 2019). The phylum *Proteobacteria*, which we found to be abundant in the preen oil of the House sparrow, was also reported to be abundant in another study on House sparrows, as well as in Dark-eyed juncos and Leach’s storm petrels (Pearce et al. 2017; Videvall et al. 2021; Whittaker et al. 2019). Similarly to our results, the class *Gammaproteobacteria* and the genus *Enhydrobacter* were found to be highly abundant in the preen gland bacteriome of House sparrows (Videvall et al. 2021). Because the House sparrow is the only urban bird in our study, we hypothesize that the different taxa found in the House sparrow may be linked to their urban environment.

### Preen oil chemical/VOC profile

The number of distinct preen oil chemical compounds ranged from 5 to 29 and consisted of alcohols, aldehydes, ketones, carboxylic acids, monoterpenes and monoterpene derivatives, unsaturated fatty acids, esters, and a few other compounds. Most of these classes of compounds were assigned as compounds with high degree of volatility (i.e. as (S)VOCs) and are frequently found in previous studies focused on passerines (Alves Soares et al. 2024). The specific aldehydes, monounsaturated fatty acids, esters, the dialkyl ketone (heptan-2-one), the phenol, and some of the monoterpenes and monoterpene derivatives that we detected are either rarely observed or have never been detected before in preen oil of passerines (Díez-Fernández et al. 2021; Shaw et al. 2011; Soini et al. 2007, 2013; Tuttle et al. 2014). However, it is important to note that due to variations in VOC sampling, storage conditions, extraction and identification methods, direct comparisons with VOC data from other studies are difficult (Alves Soares et al. 2024). We found variation in chemical/VOC profiles between the studied birds, which may reflect interspecific differences. However, due to the lack of replicates, we can not conclude that these differences are indeed reflective of species effects. Comparative studies on passerines showed interspecific differences in VOC profiles, both in the number of distinct VOCs and in classes of compounds (Soini et al. 2013; Zhang et al. 2009). Interestingly, alcohols and methyl ketones, detected in 16 out of 16 and 14 out of 16 passerine species, respectively, studied by Soini et al. (2013) (Soini et al. 2013), were only present in two species (Great reed warbler and Savi’s warbler) in our study.

Antibacterial and antifungal activity is known for many of the preen oil VOCs detected in this study, including all aldehydes (Bisignano et al. 2001; Fernando et al. 2005; Inouye et al. 2001; Lammers et al. 2021; Tao et al. 2014) and some of the alcohols (Gehrke et al. 2013), ketones (Popova et al. 2014), carboxylic acids (Chadeganipour and Haims 2001; Ganesan et al. 2022; Huang et al. 2011; Lee et al. 2021; Sahin et al. 2006; Seidel and Taylor 2004) and a few other compounds (geranyl acetone (Kubo et al. 1993), linalool (Inouye et al. 2001; Tao et al. 2014), oleic acid (Muthamil et al. 2020; Seidel and Taylor 2004), tributyl phosphate (Ferreira et al. 2010), and 2,4-ditertbutylphenol (Chawawisit et al. 2015; Zhao et al. 2020)). Several of these compounds have an antimicrobial activity against keratinolytic bacterial and fungal species (Bisignano et al. 2001; Chadeganipour and Haims 2001; Ganesan et al. 2022; Gehrke et al. 2013; Sahin et al. 2006), suggesting a potentially protective role of these preen oil compounds against feather-degrading bacteria.

Additionally, preen oil VOCs may play a role in detection by ectoparasites. Host-seeking parasites such as mosquitoes can use VOCs to detect a suitable host and the host location (Spanoudis et al. 2022; Tomás et al. 2020). On the other hand, certain VOCs can also repel parasites (Tomás et al. 2020). Several of the VOCs found in this study are known to repel or attract ornithophilic mosquitoes, including geranyl acetone, carboxylic acids, pentadecan-2-one, and aldehydes (Dormont et al. 2021; Logan et al. 2010; Mweresa et al. 2016; Puri et al. 2014; Spanoudis et al. 2022). Nonanoic acid, linalool and geranyl acetone repel *Culicoides* biting midges (González et al. 2014; Logan et al. 2009). Aldehydes, linalool, and dodecanoic acid are highly repellent or lethal against ticks and feather lice (Douglas 2008, 2013; Weldon et al. 2011; Zhu et al. 2018). Lastly, oleic acid, detected in nearly all bird species in our study, strongly repels arthropods (Yao et al. 2009). However, it should be noted that for many of these compounds, the ectoparasite repellency is strongly dose-dependent (González et al. 2014; Logan et al. 2009, 2010; Puri et al. 2014). Therefore, the lack of data on the concentrations of these VOCs in preen oil makes it difficult to determine whether the presence of these compounds in preen oil indeed plays a protective role against ectoparasites.

### Preen oil proteomic profile, and host or bacterial origins of antimicrobial proteins

For the first time, we characterized the complete proteomic profile of preen oil in passerines, where the number of proteins detected ranged from 14 to 40, and the Gene Ontology (GO) inferred biological processes associated with the proteins were dominated with proteolysis, response to oxidative stress, hydrogen peroxide catabolic process and fatty acid beta-oxidation. Fatty acid synthesis is an important function of the preen gland (Moreno-Rueda 2017). Proteins are likely to play a role in preen oil lipid synthesis (Biester et al. 2012), and proteolysis, the breakdown of proteins, plays a role in the quality control of proteins in order to regulate cellular processes (Buchberger et al. 2010). We found predominantly proteins associated with proteolysis and energy metabolism (protection against oxidative stress and fatty acid beta-oxidation) in preen oil. While only one detected protein in one species (Willow warbler) was directly related to lipid metabolism, it is possible that the proteins related to proteolysis and energy metabolism play an indirect role in fatty acid synthesis. Further experimental and physiological studies are however needed to fully understand the role of preen oil proteins in birds.

Proteins have been hypothesized to contribute to the antimicrobial activity of preen oil (Braun et al. 2018; Carneiro et al. 2020), and indeed, an earlier study detected lysozyme and immunoglobulin Y in preen oil of House sparrows (Carneiro et al. 2020). In this study, we did not find any antimicrobial proteins (e.g. bacteriocins) in our study species, except for lysozyme in the preen oil of the Long-tailed tit. This lysozyme was derived from birds and, therefore, likely synthesised by the host, not by the preen gland bacteria. Lysozymes are widespread antimicrobial enzymes that are a crucial part of the innate immune system of vertebrates (Callewaert and Michiels 2010). When present in preen oil, lysozymes may be involved in the defence of the preen gland against bacterial colonization or protect the feathers against feather-degrading bacteria (Carneiro et al. 2020).

In our study, we did not detect bacteriocins (bacterially produced antimicrobial peptides) in preen oil in any bird species, while we did detect putative bacteriocin-producing bacterial genera in all preen oil samples. It is important to note that putative bacteriocin-producing potential was inferred from presence of bacterial genera in the BACTIBASE database, not directly measured. Preen oil is hypothesized to contain bacteriocins to protect the feathers against feather-degrading bacteria, but thus far, only the Eurasian hoopoe was found to host bacteriocin-producing bacteria in their preen gland (Martín-Platero et al. 2006; Ruiz-Rodríguez et al. 2013). Potential explanations for the absence of bacteriocins in the proteomic profiles of the studied passerines are that bacteriocins are only produced under certain conditions (Braun et al. 2018; Heilbronner et al. 2021; Meade et al. 2020) and have low stability in vivo due to their susceptibility to proteolytic degradation (Meade et al. 2020). Therefore, bacteriocin detection may require other analytical methods, such as capillary electrophoresis–mass spectrometry (CE–MS) or nano LC/MS–MS preceded by a purification and filtration processes (Nandakumar and Talapatra 2014; Zou et al. 2018). The variation that we found in putative bacteriocin-producing bacteria is in line with earlier hypotheses that birds that face a higher pressure of feather-degrading bacteria on their plumage have acquired a preen oil bacteriome with higher abundances of bacteriocin-producing bacteria to protect the plumage against feather-degrading bacteria (Javůrková et al. 2019). However, in the absence of replicates, it remains uncertain whether this variation in putative bacteriocin-producing bacteria between birds is attributable to species-level differences or inter-individual variation.

### Association between preen oil bacteriome and VOCs

We have shown that the preen oil contains putative preen oil mVOC-producing bacterial genera, and that the relative abundances of these putative preen oil mVOC-producing bacterial genera varied between birds. This indicates that the preen oil bacteriome has the ability to synthesise some of the VOCs found in preen oil and that the contribution or role of the bacteriome in the production of VOCs may vary between birds. Indeed, a few previous studies have found strong indications that preen gland bacteria may produce VOCs (Martín-Vivaldi et al. 2010; Whittaker et al. 2019). However, it is important to note that this potential to produce preen oil mVOCs was inferred from a database, not measured directly. Although we did not find a statistically significant association between the preen oil bacteriome profiles and chemical profiles, this lack of association can occur if only a part of the preen oil chemical compounds is synthesised by the bacteriome and the rest by the host, which is highly probable. In addition, our sample sizes were limited to one individual per species and our chemical profile and bacteriome samples were derived from different individuals of given species, making it more difficult to directly explore the link between the bacteriome and chemical profiles. Therefore, our results on the abundances of putative preen oil mVOC producing bacterial genera and on the association between the preen oil bacteriome and chemical profiles should be interpreted with caution. Our results are however consistent with studies on dark-eyed juncos, where no covariation was detected between microbial profiles and volatile odour profiles in a field study (Whittaker et al. 2016) but found a role for preen gland bacteria in VOC production in an experimental study (Whittaker et al. 2019).

We found the highest diversity of preen oil VOC profiles and the highest relative abundances of putative preen oil mVOC-producing bacteria in the riparian bird species. Some of the compounds found exclusively in the riparian species have known antimicrobial activity, antifungal activity and mosquito and biting midge repellency (Chawawisit et al. 2015; Gehrke et al. 2013; González et al. 2014; Kubo et al. 1993; Logan et al. 2009, 2010; Popova et al. 2014; Zhao et al. 2020). The high humidity in riparian habitats may increase the prevalence of (keratinolytic) bacteria on feathers and promote the abundance of ectoparasites (Burtt and Ichida 2004; Radrova et al. 2013; Werner et al. 2020), therefore we hypothesize that the VOC profiles of the riparian species may reflect adaptive responses to habitat humidity. However, because of the limited number of species and absence of replicates, this hypothesis remains tentative and future studies with larger datasets are required to test this hypothesis.

To conclude, our findings provide preliminary insights into the preen oil bacteriome, chemical and proteomic profiles and highlight their possible associations. Further comparative and combined experimental research (e.g. adopting preen oil bacteria transplantation) with larger datasets, including more individuals per species and a broader spectrum of bird species, may help to identify the reasons for bacteriome, chemical and proteome variation between birds, which may be driven by a combination of host species traits, such as phylogeny, habitat type and other environmental factors. Moreover, the gas-phase headspace of preen oil obtained using the SPME (solid-phase micro-extraction) method is necessary for future studies, enabling more sensitive and complex profiling of preen oil VOCs.

## Supplementary Information

Below is the link to the electronic supplementary material.

Supplementary file1 (XLSX 70 KB)

Supplementary file2 (DOCX 2010 KB)

## Data Availability

The raw data, sequences, and scripts for this study are freely available online at European Nucleotide Archive (ENA) and GitHub under the following links: https://github.com/maureenbaars/preen_oil_microbiome_VOC_proteome;ENAprojectPRJEB98921

## References

[CR1] Alves Soares T, Caspers BA, Loos HM (2024) Volatile organic compounds in preen oil and feathers—a review. Biol Rev 99:1085–1099 38303487 10.1111/brv.13059

[CR2] Biester EM, Hellenbrand J, Gruber J, Hamberg M, Frentzen M (2012) Identification of avian wax synthases. BMC Biochem 13:4. 10.1186/1471-2091-13-4 22305293 10.1186/1471-2091-13-4 PMC3316144

[CR3] Bisignano G, Laganà MG, Trombetta D, Arena S, Nostro A, Uccella N, Mazzanti G, Saija A (2001) In vitro antibacterial activity of some aliphatic aldehydes from *Olea europaea* L. FEMS Microbiol Lett 198:9–13. 10.1111/j.1574-6968.2001.tb10611.x 11325546 10.1111/j.1574-6968.2001.tb10611.x

[CR4] Bodawatta KH, Schierbech SK, Petersen NR, Sam K, Bos N, Jønsson KA, Poulsen M (2020) Great tit (*Parus major*) uropygial gland microbiomes and their potential defensive roles. Front Microbiol. 10.3389/fmicb.2020.01735 32849371 10.3389/fmicb.2020.01735 PMC7401573

[CR5] Bolyen E, Rideout JR, Dillon MR et al (2019) Reproducible, interactive, scalable and extensible microbiome data science using QIIME 2. Nat Biotechnol 37:852–857. 10.1038/s41587-019-0209-9 31341288 10.1038/s41587-019-0209-9 PMC7015180

[CR6] Braun MS, Sporer F, Zimmermann S, Wink M (2018) Birds, feather-degrading bacteria and preen glands: the antimicrobial activity of preen gland secretions from turkeys (*Meleagris gallopavo*) is amplified by keratinase. FEMS Microbiol Ecol. 10.1093/femsec/fiy117 29901706 10.1093/femsec/fiy117

[CR7] Buchberger A, Bukau B, Sommer T (2010) Protein quality control in the cytosol and the endoplasmic reticulum: brothers in arms. Mol Cell 40:238–252. 10.1016/j.molcel.2010.10.001 20965419 10.1016/j.molcel.2010.10.001

[CR8] Burger BV, Reiter B, Borzyk O, du Plessis MA (2004) Avian exocrine secretions. I. Chemical characterization of the volatile fraction of the uropygial secretion of the green woodhoopoe, *Phoeniculus purpureus*. J Chem Ecol 30:1603–1611. 10.1023/B:JOEC.0000042071.65335.f3 15537162 10.1023/b:joec.0000042071.65335.f3

[CR10] Burtt EH Jr, Ichida JM (2004) Gloger’s rule, feather-degrading bacteria, and color variation among song sparrows. Condor 106:681–686. 10.1093/condor/106.3.681

[CR12] Callahan BJ, McMurdie PJ, Rosen MJ, Han AW, Johnson AJA, Holmes SP (2016) DADA2: high-resolution sample inference from Illumina amplicon data. Nat Methods 13:581–583 27214047 10.1038/nmeth.3869 PMC4927377

[CR13] Callewaert L, Michiels CW (2010) Lysozymes in the animal kingdom. J Biosci (Bangalore) 35:127–160. 10.1007/s12038-010-0015-5 10.1007/s12038-010-0015-5 20413917

[CR15] Carneiro D, Czirják GÁ, Rowe M (2020) Innate and adaptive immune proteins in the preen gland secretions of male house sparrows. J Avian Biol. 10.1111/jav.02556

[CR16] Chadeganipour M, Haims A (2001) Antifungal activities of pelargonic and capric acid on *Microsporum gypseum*. Mycoses 44:109–112 11413921 10.1046/j.1439-0507.2001.00609.x

[CR17] Chawawisit K, Bhoopong P, Phupong W, Lertcanawanichakul M (2015) 2, 4-Di-tert-butylphenol, the bioactive compound produced by *Streptomyces* sp. KB1. J Appl Pharm Sci 5(Suppl 3):007–012

[CR18] Czirják GÁ, Pap PL, Vágási CI, Giraudeau M, Mureşan C, Mirleau P, Heeb P (2013) Preen gland removal increases plumage bacterial load but not that of feather-degrading bacteria. Naturwissenschaften 100:145–151 23288399 10.1007/s00114-012-1005-2

[CR19] Díez-Fernández A, Martínez-de la Puente J, Martín J, Gangoso L, López P, Soriguer R, Figuerola J (2021) Sex and age, but not blood parasite infection nor habitat, affect the composition of the uropygial gland secretions in European blackbirds. J Avian Biol. 10.1111/jav.02630

[CR21] Directive 2010/75/EU (2010) Directive 2010/75/EU of the European Parliament and of the Council of 24 November 2010 on industrial emissions (integrated pollution prevention and control). https://eur-lex.europa.eu/eli/dir/2010/75/oj/eng

[CR22] Dormont L, Mulatier M, Carrasco D, Cohuet A (2021) Mosquito attractants. J Chem Ecol 47:351–393. 10.1007/s10886-021-01261-2 33725235 10.1007/s10886-021-01261-2

[CR23] Douglas HD (2008) Prenuptial perfume: Alloanointing in the social rituals of the crested auklet (*Aethia cristatella*) and the transfer of arthropod deterrents. Naturwissenschaften 95:45–53. 10.1007/s00114-007-0294-3 17703279 10.1007/s00114-007-0294-3

[CR24] Douglas HD (2013) Colonial seabird’s paralytic perfume slows lice down: an opportunity for parasite-mediated selection? Int J Parasitol 43:399–407 23399419 10.1016/j.ijpara.2013.01.004

[CR25] Edgar RC, Haas BJ, Clemente JC, Quince C, Knight R (2011) Uchime improves sensitivity and speed of chimera detection. Bioinformatics 27:2194–2200 21700674 10.1093/bioinformatics/btr381 PMC3150044

[CR26] Engel K, Sauer J, Jünemann S, Winkler A, Wibberg D, Kalinowski J, Tauch A, Caspers BA (2018) Individual- and species-specific skin microbiomes in three different estrildid finch species revealed by 16S amplicon sequencing. Microb Ecol 76:518–529. 10.1007/s00248-017-1130-8 29282519 10.1007/s00248-017-1130-8

[CR27] Fernando WGD, Ramarathnam R, Krishnamoorthy AS, Savchuk SC (2005) Identification and use of potential bacterial organic antifungal volatiles in biocontrol. Soil Biol Biochem 37:955–964

[CR28] Ferreira JP, Alves D, Neves O, Silva J, Gibbs PA, Teixeira PC (2010) Effects of the components of two antimicrobial emulsions on food-borne pathogens. Food Control 21:227–230

[CR29] Fliegerova K, Tapio I, Bonin A et al (2014) Effect of DNA extraction and sample preservation method on rumen bacterial population. Anaerobe 29:80–84 24125910 10.1016/j.anaerobe.2013.09.015

[CR30] Ganesan T, Subban M, Christopher Leslee DB, Kuppannan SB, Seedevi P (2022) Structural characterization of n-hexadecanoic acid from the leaves of *Ipomoea eriocarpa* and its antioxidant and antibacterial activities. Biomass Convers Biorefin. 10.1007/s13399-022-03576-w

[CR31] Gehrke ITS, Neto AT, Pedroso M, Mostardeiro CP, Da Cruz IBM, Silva UF, Ilha V, Dalcol II, Morel AF (2013) Antimicrobial activity of *Schinus lentiscifolius* (*Anacardiaceae*). J Ethnopharmacol 148:486–491 23684720 10.1016/j.jep.2013.04.043

[CR32] Giraudeau M, Czirják GÁ, Duval C, Bretagnolle V, Gutierrez C, Guillon N, Heeb P (2013) Effect of preen oil on plumage bacteria: an experimental test with the mallard. Behav Processes 92:1–5 22940115 10.1016/j.beproc.2012.08.001

[CR34] González m, Venter gJ, López s, Iturrondobeitia jC, Goldarazena a (2014) Laboratory and field evaluations of chemical and plant-derived potential repellents against *Culicoides* biting midges in northern Spain. Med Vet Entomol 28:421–431 25079042 10.1111/mve.12081

[CR35] Grieves LA, Gloor GB, Kelly TR, Bernards MA, MacDougall-Shackleton EA (2021) Preen gland microbiota of songbirds differ across populations but not sexes. J Anim Ecol 90:2202–2212 34002375 10.1111/1365-2656.13531

[CR107] Grieves LA, Gilles M, Cuthill IC, Székely T, MacDougall-Shackleton EA, Caspers BA (2022) Olfactory camouflage and communication in birds. Biol Rev 97:1193–1209. 10.1111/brv.12837 35128775 10.1111/brv.12837

[CR108] Grieves LA, Gloor GB (2025) Uropygial gland microbiota of nearctic−neotropical migrants vary with season and migration distance. Animal Microbiome 7:11. 10.1186/s42523-024-00367-8 39885562 10.1186/s42523-024-00367-8 PMC11780944

[CR36] Hammami R, Zouhir A, Le Lay C, Ben Hamida J, Fliss I (2010) BACTIBASE second release: a database and tool platform for bacteriocin characterization. BMC Microbiol 10:22. 10.1186/1471-2180-10-22 20105292 10.1186/1471-2180-10-22 PMC2824694

[CR37] Heilbronner S, Krismer B, Brötz-Oesterhelt H, Peschel A (2021) The microbiome-shaping roles of bacteriocins. Nat Rev Microbiol 19:726–739. 10.1038/s41579-021-00569-w 34075213 10.1038/s41579-021-00569-w

[CR38] Huang CB, Alimova Y, Myers TM, Ebersole JL (2011) Short- and medium-chain fatty acids exhibit antimicrobial activity for oral microorganisms. Arch Oral Biol 56:650–654 21333271 10.1016/j.archoralbio.2011.01.011 PMC3119748

[CR39] Inouye S, Takizawa T, Yamaguchi H (2001) Antibacterial activity of essential oils and their major constituents against respiratory tract pathogens by gaseous contact. J Antimicrob Chemother 47:565–573. 10.1093/jac/47.5.565 11328766 10.1093/jac/47.5.565

[CR40] Jackson DA (1995) PROTEST: a PROcrustean randomization TEST of community environment concordance. Ecoscience 2:297–303. 10.1080/11956860.1995.11682297

[CR41] Jacob J, Ziswiler V (1982) The uropygial gland. Avian Biol 6:199–324

[CR42] Javůrková VG, Kreisinger J, Procházka P, Požgayová M, Ševčíková K, Brlík V, Adamík P, Heneberg P, Porkert J (2019) Unveiled feather microcosm: feather microbiota of passerine birds is closely associated with host species identity and bacteriocin-producing bacteria. ISME J 13:2363–2376. 10.1038/s41396-019-0438-4 31127178 10.1038/s41396-019-0438-4 PMC6775979

[CR43] Jiang H, Lei R, Ding S-W, Zhu S (2014) Skewer: a fast and accurate adapter trimmer for next-generation sequencing paired-end reads. BMC Bioinformatics 15:182. 10.1186/1471-2105-15-182 24925680 10.1186/1471-2105-15-182 PMC4074385

[CR44] Kemmler E, Lemfack MC, Goede A, Gallo K, Toguem SM, Ahmed W, Millberg I, Preissner S, Piechulla B, Preissner R (2025) mVOC 4.0: a database of microbial volatiles. Nucleic Acids Res 53:D1692–D1696 39475188 10.1093/nar/gkae961 PMC11701663

[CR45] Krassowski M (2021) ComplexUpset: create complex UpSet plots using “ggplot2” components. R package version 1.3.3

[CR46] Kubo I, Muroi H, Kubo A (1993) Antibacterial activity of long-chain alcohols against *Streptococcus mutans*. J Agric Food Chem 41:2447–2450. 10.1021/jf00036a045

[CR47] Lammers A, Zweers H, Sandfeld T, Bilde T, Garbeva P, Schramm A, Lalk M (2021) Antimicrobial compounds in the volatilome of social spider communities. Front Microbiol. 10.3389/fmicb.2021.700693 34504476 10.3389/fmicb.2021.700693 PMC8422909

[CR48] Law-Brown J (2001) Chemical defence in the red-billed wood hoopoe: *phoeniculus purpureus*. University of Cape Town

[CR49] Lee J-H, Kim Y-G, Khadke SK, Lee J (2021) Antibiofilm and antifungal activities of medium-chain fatty acids against *Candida albicans* via mimicking of the quorum-sensing molecule farnesol. Microb Biotechnol 14:1353–1366 33252828 10.1111/1751-7915.13710 PMC8313291

[CR50] Liu X (2022) Understanding semi-volatile organic compounds in indoor dust. Indoor Built Environ 31:291–298 35221787 10.1177/1420326x211070859 PMC8879700

[CR51] Logan JG, Seal NJ, Cook JI, Stanczyk NM, Birkett MA, Clark SJ, Pickett JA (2009) Identification of human-derived volatile chemicals that interfere with attraction of the scottish biting midge and their potential use as repellents. J Med Entomol 46(2):208–219. 10.1603/033.046.0205 19351071 10.1603/033.046.0205

[CR52] Logan JG, Stanczyk NM, Hassanali A, Kemei J, Santana AEG, Ribeiro KAL, Pickett JA, Mordue AJ (2010) Arm-in-cage testing of natural human-derived mosquito repellents. Malar J 9:239. 10.1186/1475-2875-9-239 20727149 10.1186/1475-2875-9-239 PMC2931528

[CR53] Magallanes S, Møller AP, García-Longoria L, de Lope F, Marzal A (2016) Volume and antimicrobial activity of secretions of the uropygial gland are correlated with malaria infection in house sparrows. Parasit Vectors 9:232. 10.1186/s13071-016-1512-7 27114098 10.1186/s13071-016-1512-7 PMC4845389

[CR55] Martínez-Renau E, Mazorra-Alonso M, Ruiz-Castellano C, Martín-Vivaldi M, Martín-Platero AM, Barón MD, Soler JJ (2022) Microbial infection risk predicts antimicrobial potential of avian symbionts. Front Microbiol. 10.3389/fmicb.2022.1010961 36478864 10.3389/fmicb.2022.1010961 PMC9719979

[CR56] Martín-Platero AM, Valdivia E, Ruíz-Rodríguez M, Soler JJ, Martín-Vivaldi M, Maqueda M, Martínez-Bueno M (2006) Characterization of antimicrobial substances produced by *Enterococcus faecalis* MRR 10–3, isolated from the uropygial gland of the Hoopoe (*Upupa epops*). Appl Environ Microbiol 72:4245–4249 16751538 10.1128/AEM.02940-05 PMC1489579

[CR57] Martín-Vivaldi M, Peña A, Peralta-Sánchez JM, Sánchez L, Ananou S, Ruiz-Rodríguez M, Soler JJ (2010) Antimicrobial chemicals in hoopoe preen secretions are produced by symbiotic bacteria. Proc R Soc B 277:123–130 19812087 10.1098/rspb.2009.1377 PMC2842625

[CR59] Meade E, Slattery MA, Garvey M (2020) Bacteriocins, potent antimicrobial peptides and the fight against multi drug resistant species: resistance is futile? Antibiotics 9:32 31963311 10.3390/antibiotics9010032 PMC7168330

[CR60] Møller AP, Erritzøe J, Rózsa L (2010) Ectoparasites, uropygial glands and hatching success in birds. Oecologia 163:303–311. 10.1007/s00442-009-1548-x 20043177 10.1007/s00442-009-1548-x

[CR61] Moreno-Rueda G (2011) House sparrows *Passer domesticus* with larger uropygial glands show reduced feather wear. Ibis 153:195–198

[CR62] Moreno-Rueda G (2017) Preen oil and bird fitness: a critical review of the evidence. Biol Rev 92:2131–2143 28231637 10.1111/brv.12324

[CR63] Muthamil S, Prasath KG, Priya A, Precilla P, Pandian SK (2020) Global proteomic analysis deciphers the mechanism of action of plant derived oleic acid against *Candida albicans* virulence and biofilm formation. Sci Rep 10:5113. 10.1038/s41598-020-61918-y 32198447 10.1038/s41598-020-61918-y PMC7083969

[CR64] Mweresa CK, Mukabana WR, Omusula P, Otieno B, Van Loon JJA, Takken W (2016) Enhancing attraction of African malaria vectors to a synthetic odor blend. J Chem Ecol 42:508–516. 10.1007/s10886-016-0711-1 27349651 10.1007/s10886-016-0711-1

[CR65] Nandakumar R, Talapatra K (2014) Quantitative profiling of bacteriocins present in dairy-free probiotic preparations of *Lactobacillus acidophilus* by nanoliquid chromatography-tandem mass spectrometry. J Dairy Sci 97:1999–2008 24565320 10.3168/jds.2013-7470

[CR66] Oksanen J, Simpson G, Blanchet F et al. (2022) Vegan: community ecology package, R Package Version 2.6-4

[CR67] Pearce DS, Hoover BA, Jennings S, Nevitt GA, Docherty KM (2017) Morphological and genetic factors shape the microbiome of a seabird species (*Oceanodroma leucorhoa*) more than environmental and social factors. Microbiome 5:146. 10.1186/s40168-017-0365-4 29084611 10.1186/s40168-017-0365-4 PMC5663041

[CR68] Peres-Neto PR, Jackson DA (2001) How well do multivariate data sets match? The advantages of a Procrustean superimposition approach over the Mantel test. Oecologia 129:169–178. 10.1007/s004420100720 28547594 10.1007/s004420100720

[CR69] Popova AA, Koksharova OA, Lipasova VA, Zaitseva JV, Katkova-Zhukotskaya OA, Eremina SI, Mironov AS, Chernin LS, Khmel IA (2014) Inhibitory and toxic effects of volatiles emitted by strains of *Pseudomonas* and *Serratia* on growth and survival of selected microorganisms, *Caenorhabditis elegans*, and *Drosophila melanogaster*. BioMed Res Int 2014:125704. 10.1155/2014/125704 25006575 10.1155/2014/125704 PMC4071779

[CR70] Potier S, Besnard MM, Schikorski D, Buatois B, Duriez O, Gabirot M, Leclaire S, Bonadonna F (2018) Preen oil chemical composition encodes individuality, seasonal variation and kinship in black kites *Milvus migrans*. J Avian Biol 49:e01728

[CR71] Puri SN, Mendki MJ, Sukumaran D, Ganesan K, Prakash S, Sekhar K (2014) Electroantennogram and behavioral responses of *Culex quinquefasciatus (Diptera*: *Culicidae*) females to chemicals found in human skin emanations. J Med Entomol 43:207–213. 10.1093/jmedent/43.2.207 10.1603/0022-2585(2006)043[0207:eabroc]2.0.co;2 16619600

[CR72] Quast C, Pruesse E, Yilmaz P, Gerken J, Schweer T, Yarza P, Peplies J, Glöckner FO (2012) The SILVA ribosomal RNA gene database project: improved data processing and web-based tools. Nucleic Acids Res 41:D590–D596. 10.1093/nar/gks1219 23193283 10.1093/nar/gks1219 PMC3531112

[CR73] Radrova J, Seblova V, Votypka J (2013) Feeding behavior and spatial distribution of *Culex* mosquitoes (*Diptera*: *Culicidae)* in wetland areas of the Czech Republic. J Med Entomol 50:1097–1104. 10.1603/ME13029 24180115 10.1603/me13029

[CR74] Rappsilber J, Mann M, Ishihama Y (2007) Protocol for micro-purification, enrichment, pre-fractionation and storage of peptides for proteomics using stagetips. Nat Protoc 2:1896–1906. 10.1038/nprot.2007.261 17703201 10.1038/nprot.2007.261

[CR75] Rodríguez-Ruano SM, Martín-Vivaldi M, Peralta-Sánchez JM, García-Martín AB, Martínez-García Á, Soler JJ, Valdivia E, Martínez-Bueno M (2018) Seasonal and sexual differences in the microbiota of the Hoopoe uropygial secretion. Genes 9:407 30103505 10.3390/genes9080407 PMC6115775

[CR76] Roggenbuck M, Bærholm Schnell I, Blom N, Bælum J, Bertelsen MF, Sicheritz-Pontén T, Sørensen SJ, Gilbert MTP, Graves GR, Hansen LH (2014) The microbiome of New World vultures. Nat Commun 5:5498. 10.1038/ncomms6498 25423494 10.1038/ncomms6498

[CR77] Ruiz-Rodríguez M, Valdivia E, Soler JJ, Martín-Vivaldi M, Martín-Platero AM, Martínez-Bueno M (2009) Symbiotic bacteria living in the hoopoe’s uropygial gland prevent feather degradation. J Exp Biol 212:3621–3626. 10.1242/jeb.031336 19880722 10.1242/jeb.031336

[CR78] Ruiz-Rodríguez M, Valdivia E, Martín-Vivaldi M, Martín-Platero AM, Martínez-Bueno M, Méndez M, Peralta-Sánchez JM, Soler JJ (2012) Antimicrobial activity and genetic profile of *Enteroccoci* isolated from hoopoes uropygial gland. PLoS ONE 7:e41843. 10.1371/journal.pone.0041843 22911858 10.1371/journal.pone.0041843 PMC3404078

[CR79] Ruiz-Rodríguez M, Martínez-Bueno M, Martín-Vivaldi M, Valdivia E, Soler JJ (2013) Bacteriocins with a broader antimicrobial spectrum prevail in enterococcal symbionts isolated from the hoopoe’s uropygial gland. FEMS Microbiol Ecol 85:495–502. 10.1111/1574-6941.12138 23621827 10.1111/1574-6941.12138

[CR80] Ruiz-Rodríguez M, Tomás G, Martín-Gálvez D, Ruiz-Castellano C, Soler JJ (2015) Bacteria and the evolution of honest signals. The case of ornamental throat feathers in spotless starlings. Funct Ecol 29:701–709. 10.1111/1365-2435.12376

[CR81] Sahin N, Kula I, Erdogan Y (2006) Investigation of antimicrobial activities of nonanoic acid derivatives. Fresenius Environ Bull 15:141–143

[CR82] Salter SJ, Cox MJ, Turek EM, Calus ST, Cookson WO, Moffatt MF, Turner P, Parkhill J, Loman NJ, Walker AW (2014) Reagent and laboratory contamination can critically impact sequence-based microbiome analyses. BMC Biol 12:87. 10.1186/s12915-014-0087-z 25387460 10.1186/s12915-014-0087-z PMC4228153

[CR83] Seidel V, Taylor PW (2004) In vitro activity of extracts and constituents of *Pelagonium* against rapidly growing mycobacteria. Int J Antimicrob Agents 23:613–619 15194133 10.1016/j.ijantimicag.2003.11.008

[CR84] Shaw CL, Rutter JE, Austin AL, Garvin MC, Whelan RJ (2011) Volatile and semivolatile compounds in Gray Catbird uropygial secretions vary with age and between breeding and wintering grounds. J Chem Ecol 37:329–339. 10.1007/s10886-011-9931-6 21424249 10.1007/s10886-011-9931-6

[CR85] Shawkey MD, Pillai SR, Hill GE (2003) Chemical warfare? Effects of uropygial oil on feather-degrading bacteria. J Avian Biol 34:345–349

[CR86] Soini HA, Schrock SE, Bruce KE, Wiesler D, Ketterson ED, Novotny MV (2007) Seasonal variation in volatile compound profiles of preen gland secretions of the dark-eyed junco (*Junco hyemalis*). J Chem Ecol 33:183–198. 10.1007/s10886-006-9210-0 17146717 10.1007/s10886-006-9210-0

[CR87] Soini HA, Whittaker DJ, Wiesler D, Ketterson ED, Novotny MV (2013) Chemosignaling diversity in songbirds: chromatographic profiling of preen oil volatiles in different species. J Chromatogr 1317:186–192 10.1016/j.chroma.2013.08.006 23998336

[CR88] Soler JJ, Martín-Vivaldi M, Ruiz-Rodríguez M, Valdivia E, Martín-Platero AM, Martínez-Bueno M, Peralta-Sánchez JM, Méndez M (2008) Symbiotic association between hoopoes and antibiotic-producing bacteria that live in their uropygial gland. Funct Ecol 22:864–871

[CR110] Soler JJ, Barón MD, Martínez-Renau E, Zhang L, Liang W, Martín-Vivaldi M (2024) Nesting hoopoes cultivate in their uropygial gland the microbial symbionts with the highest antimicrobial capacity. Scientific Reports 14:30797. 10.1038/s41598-024-81062-1 39730533 10.1038/s41598-024-81062-1 PMC11681103

[CR89] Soudy M, Anwar AM, Ahmed EA, Osama A, Ezzeldin S, Mahgoub S, Magdeldin S (2020) UniprotR: retrieving and visualizing protein sequence and functional information from universal protein resource (UniProt knowledgebase). J Proteomics 213:103613 31843688 10.1016/j.jprot.2019.103613

[CR90] Spanoudis CG, Wondwosen B, Isberg E, Andreadis SS, Kline DL, Birgersson G, Ignell R (2022) The chemical code for attracting *Culex* mosquitoes. Front Ecol Evol. 10.3389/fevo.2022.930665

[CR111] Šta̕stný K, Hudec K (2011) Fauna ČR Ptáci 3/II. 2nd edn. Academia (Středisko spol. činností AV ČR, v. v. i.), Prague

[CR91] Tao N, Jia L, Zhou H (2014) Anti-fungal activity of *Citrus reticulata* Blanco essential oil against *Penicillium italicum* and *Penicillium digitatum*. Food Chem 153:265–271 24491729 10.1016/j.foodchem.2013.12.070

[CR92] Tomás G, Zamora-Muñoz C, Martín-Vivaldi M, Barón MD, Ruiz-Castellano C, Soler JJ (2020) Effects of chemical and auditory cues of Hoopoes (*Upupa epops*) in repellence and attraction of blood-feeding flies. Front Ecol Evol. 10.3389/fevo.2020.579667

[CR93] Tuttle EM, Sebastian PJ, Posto AL, Soini HA, Novotny MV, Gonser RA (2014) Variation in preen oil composition pertaining to season, sex, and genotype in the polymorphic white-throated sparrow. J Chem Ecol 40:1025–1038. 10.1007/s10886-014-0493-2 25236380 10.1007/s10886-014-0493-2

[CR94] van Veelen HPJ, Falcao Salles J, Tieleman BI (2017) Multi-level comparisons of cloacal, skin, feather and nest-associated microbiota suggest considerable influence of horizontal acquisition on the microbiota assembly of sympatric woodlarks and skylarks. Microbiome 5:156. 10.1186/s40168-017-0371-6 29191217 10.1186/s40168-017-0371-6 PMC5709917

[CR95] Videvall E, Marzal A, Magallanes S, Fleischer RC, Espinoza K, García-Longoria L (2021) The uropygial gland microbiome of house sparrows with malaria infection. J Avian Biol. 10.1111/jav.02686

[CR96] Wang Q, Garrity GM, Tiedje JM, Cole JR (2007) Naïve bayesian classifier for rapid assignment of rRNA sequences into the new bacterial taxonomy. Appl Environ Microbiol 73:5261–5267 17586664 10.1128/AEM.00062-07 PMC1950982

[CR97] Weldon PJ, Carroll JF, Kramer M, Bedoukian RH, Coleman RE, Bernier UR (2011) Anointing chemicals and hematophagous arthropods: responses by ticks and mosquitoes to citrus (*Rutaceae*) peel exudates and monoterpene components. J Chem Ecol 37:348–359. 10.1007/s10886-011-9922-7 21409496 10.1007/s10886-011-9922-7

[CR98] Werner D, Groschupp S, Bauer C, Kampen H (2020) Breeding habitat preferences of major *Culicoides* species (*Diptera*: *Ceratopogonidae*) in Germany. Int J Environ Res Public Health 17(14):5000 32664561 10.3390/ijerph17145000 PMC7400431

[CR99] Whittaker DJ, Gerlach NM, Slowinski SP, Corcoran KP, Winters AD, Soini HA, Novotny MV, Ketterson ED, Theis KR (2016) Social environment has a primary influence on the microbial and odor profiles of a chemically signaling songbird. Front Ecol Evol. 10.3389/fevo.2016.00090

[CR100] Whittaker DJ, Slowinski SP, Greenberg JM, Alian O, Winters AD, Ahmad MM, Burrell MJE, Soini HA, Novotny MV, Ketterson ED, Theis KR (2019) Experimental evidence that symbiotic bacteria produce chemical cues in a songbird. J Exp Biol. 10.1242/jeb.202978 31537652 10.1242/jeb.202978

[CR109] Whittaker DJ, Hagelin JC (2021) Female-Based Patterns and Social Function in Avian Chemical Communication. J Chem Ecol 47:43–62. 10.1007/s10886-020-01230-1 33103230 10.1007/s10886-020-01230-1

[CR101] Wickham H (2016) ggplot2: elegant graphics for data analysis. Springer, New York. 10.1007/978-3-319-24277-4

[CR102] Yao M, Rosenfeld J, Attridge S, Sidhu S, Aksenov V, Rollo CD (2009) The ancient chemistry of avoiding risks of predation and disease. Evol Biol 36:267–281. 10.1007/s11692-009-9069-4

[CR103] Zhang J-X, Sun L, Zuo M-X (2009) Uropygial gland volatiles may code for olfactory information about sex, individual, and species in Bengalese finches *Lonchura striata*. Curr Zool 55:357–365. 10.1093/czoolo/55.5.357

[CR104] Zhao F, Wang P, Lucardi RD, Su Z, Li S (2020) Natural sources and bioactivities of 2,4-Di-Tert-Butylphenol and its analogs. Toxins 12:35 31935944 10.3390/toxins12010035 PMC7020479

[CR105] Zhu JJ, Cermak SC, Kenar JA et al (2018) Better than DEET repellent compounds derived from coconut oil. Sci Rep 8:14053. 10.1038/s41598-018-32373-7 30232355 10.1038/s41598-018-32373-7 PMC6145915

[CR106] Zou J, Jiang H, Cheng H, Fang J, Huang G (2018) Strategies for screening, purification and characterization of bacteriocins. Int J Biol Macromol 117:781–789 29870810 10.1016/j.ijbiomac.2018.05.233

