# Supplementary material for: Exploring the associations between preen oil bacterial, chemical and proteomic profiles of passerines
Source: Antonie Van Leeuwenhoek. 2025 Oct 16;118(11):173. doi: 10.1007/s10482-025-02182-w (PMC12531318; doi:10.1007/s10482-025-02182-w)
Supplement: Supplementary file 2 — Supplementary file2 (DOCX 2010 KB) [file 10482_2025_2182_MOESM2_ESM.docx]

**Exploring The Associations Between Preen Oil Bacterial, Chemical And Proteomic Profiles Of Passerines**

*Antonie van Leeuwenhoek*

I. Maureen Baars^1*^, Jakub Mrázek ^2^, Jakub Kreisinger^3^, Ivan Mikšík^4^, Maurine W. Dietz^1^, Joana Falcao Salles^1^, B. Irene Tieleman^1^, Veronika Gvoždíková Javůrková^1,5*^

^1^ *Groningen Institute for Evolutionary Life Sciences, University of Groningen, Nijenborgh 7, 9747 AG, Groningen, Netherlands.*

*^2^ Institute of Animal Physiology and Genetics, Czech Academy of Sciences, Vídeňská*

*1083,160 00, Prague-Krč, Czech Republic*

^3^ *Faculty of Science, Department of Zoology, Charles University, Viničná 7, 128 44, Prague, Czech Republic*

*^4^ Department of Analytical Chemistry, Faculty of Chemical Technology, University of Pardubice, Studentská 573, 532 10 Pardubice, Czech Republic*

*^5^ Institute of Vertebrate Biology of the Czech Academy of Sciences, Květná 8, 603 65*

*Brno, Czech Republic*

* Corresponding authors:

I. Maureen Baars: [i.m.baars@rug.nl](mailto:i.m.baars@rug.nl)

Veronika Gvoždíková Javůrková: [v.gvozdikova.javurkova@rug.nl](mailto:v.gvozdikova.javurkova@rug.nl)

**Table S1.** Overview of passerine species sampled in this study for chemical, proteomic and bacteriome profiling of preen oil. List of passerine species used in this study for A) chemical profiling, B) proteomic profiling and C) bacterial community profiling with information on sex, age, date of capture, site and geographic location, and the breeding (laying eggs) peak date in the Czech Republic {Šta̕stný, 2011 #460}.

| **A) chemical profiling** | | |  |  |  |  |  |  |  |  |
| --- | --- | --- | --- | --- | --- | --- | --- | --- | --- | --- |
| **Sample ID** | **Species name** | **Species Latin name** | **Analysis** | **Sex** | **Age** | **Capture date** | **Locality** | **Latitude** | **Longitude** | **Breeding peak date** |
| N723712 | European Nuthatch | *Sitta europaea* | VOC | M | 1Y+ | 16.4.2015 | pond Hodov Valdíkov | 49.24235 | 15.99054 | 15 - 30.4 |
| ZA35003 | Great Reed Warbler | *Acrocephalus arundinaceus* | VOC | M | 1Y+ | 22.4.2016 | Mutěnické ponds | 48.9046 | 17.04934 | 10 - 30.5 |
| J89374 | Willow Warbler | *Phylloscopus trochilus* | VOC | M | 1Y+ | 28.4.2015 | Běleč nad Orlicí | 50.17433 | 15.95526 | 10 - 30.5 |
| TS29041 | Savi´s Warbler | *Locustella luscinioides* | VOC | M | 1Y+ | 21.4.2016 | Mutěnické ponds | 48.8954 | 17.06071 | 10 - 30.5 |
| J89361 | Long-tailed Tit | *Aegithalos caudatus* | VOC | M | 1Y+ | 28.3.2015 | Budišov park | 49.2761 | 16.012498 | 15 - 30.4 |
| TP29927 | Common Redstart | *Phoenicurus phoenicurus* | VOC | M | 1Y+ | 30.4.2015 | Běleč nad Orlicí | 50.17433 | 15.95526 | 10 - 20.5 |
| N723731 | House Sparrow | *Passer domesticus* | VOC | M | 1Y+ | 27.3.2016 | Hladké Životice_grocery | 49.68442 | 17.95407 | 20.4 - 10.5 |
| **B) proteomic profiling** | | |  |  |  |  |  |  |  |  |
| **Sample ID** | **Species name** | **Species Latin name** | **Analysis** | **Sex** | **Age** | **Capture date** | **Locality** | **Latitude** | **Longitude** | **Breeding peak date** |
| ZA35998 | Great Reed Warbler | *Acrocephalus arundinaceus* | proteomic | M | 1Y+ | 25.4.2016 | Mutěnické ponds | 48.9046 | 17.04934 | 10 - 30.5 |
| JA0118 | Long-tailed Tit | *Aegithalos caudatus* | proteomic | M | 1Y+ | 12.4.2015 | Olomouc Smetanovy park | 49.58925 | 17.2488 | 15 - 30.4 |
| J89373 | Willow Warbler | *Phylloscopus trochilus* | proteomic | M | 1Y+ | 28.4.2015 | Běleč nad Orlicí | 50.17433 | 15.95526 | 10 - 30.5 |
| 8_ S605052 | Sand Martin | *Riparia riparia* | proteomic | F | 3Y | 29.5.2015 | Liteň sand plant | 49.895978 | 14.137203 | 20 - 30.5 |
| 9_RRB9K | Sand Martin | *Riparia riparia* | proteomic | F | 1Y+ | 29.5.2015 | Liteň sand plant | 49.895978 | 14.137203 | 20 - 30.5 |
| **C) bacteriome profiling** | | |  |  |  |  |  |  |  |  |
| **Sample ID** | **Species name** | **Species Latin name** | **Analysis** | **Sex** | **Age** | **Capture date** | **Locality** | **Latitude** | **Longitude** | **Breeding peak date** |
| N_723713 | European Nuthatch | *Sitta europaea* | bacteriome | M | 1Y+ | 16.4.2015 | pond Hodov Valdíkov | 49.24235 | 15.99054 | 15 - 30.4 |
| Liteň 6 = 86 | Sand Martin | *Riparia riparia* | bacteriome | F | 1Y+ | 29.5.2015 | Liteň sand plant | 49.895978 | 14.137203 | 20 - 30.5 |
| ZA43517 | Great Reed Warbler | *Acrocephalus arundinaceus* | bacteriome | M | 1Y+ | 29.4.2016 | Mutěnické ponds | 48.8954 | 17.06071 | 10 - 30.5 |
| J89375 | Willow Warbler | *Phylloscopus trochilus* | bacteriome | M | 1Y+ | 28.4.2015 | Běleč nad Orlicí | 50.17433 | 15.95526 | 10 - 30.5 |
| TS29198 | Savi´s Warbler | *Locustella luscinioides* | bacteriome | M | 1Y+ | 21.4.2016 | Mutěnické ponds | 48.8954 | 17.06071 | 10 - 30.5 |
| J89392 | Long-tailed Tit | *Aegithalos caudatus* | bacteriome | M | 1Y+ | 25.3.2016 | Suchdol nad Odrou | 49.64528 | 17.95453 | 15 - 30.4 |
| TP63745 | Common Redstart | *Phoenicurus phoenicurus* | bacteriome | M | 1Y+ | 29.4.2015 | Běleč nad Orlicí | 50.17433 | 15.95526 | 10 - 20.5 |
| N723739 | House Sparrow | *Passer domesticus* | bacteriome | M | 1Y+ | 27.3.2016 | Hladké Životice_grocery | 49.68442 | 17.95407 | 20.4 - 10.5 |

**
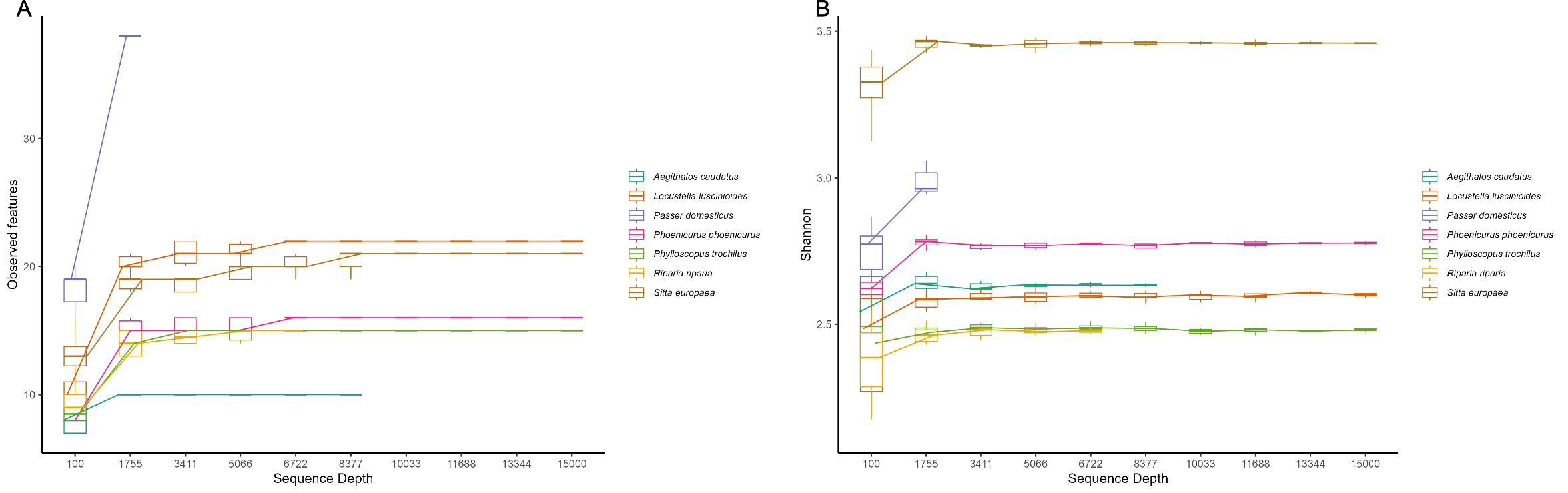
**

**Fig. S1** Alpha rarefaction curves for A) observed features (ASVs) and B) Shannon diversity. Sequencing depth = number of reads per sample. Different species are denoted by different colours


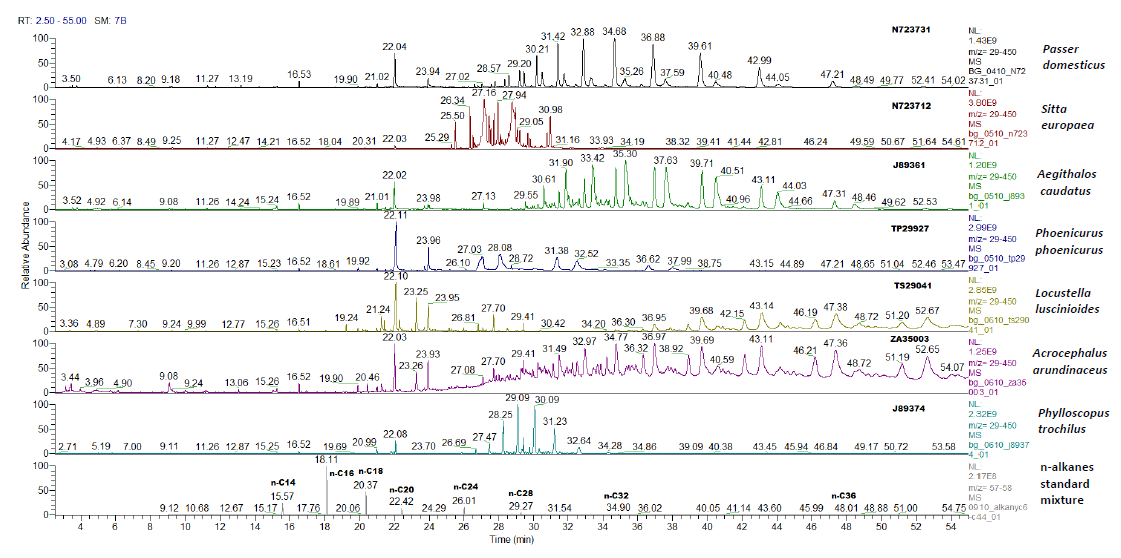
 **A)**

**B)**


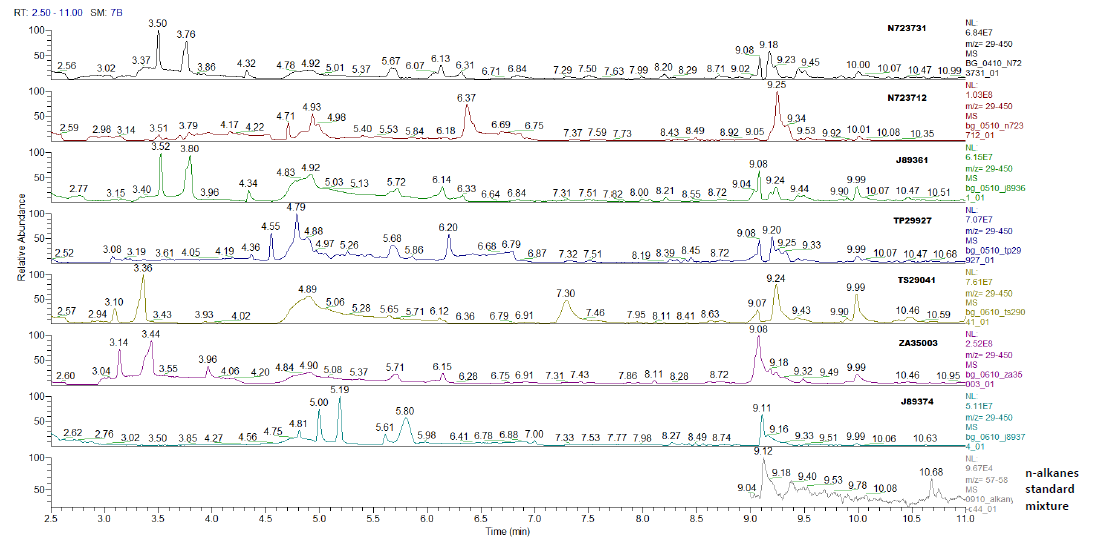


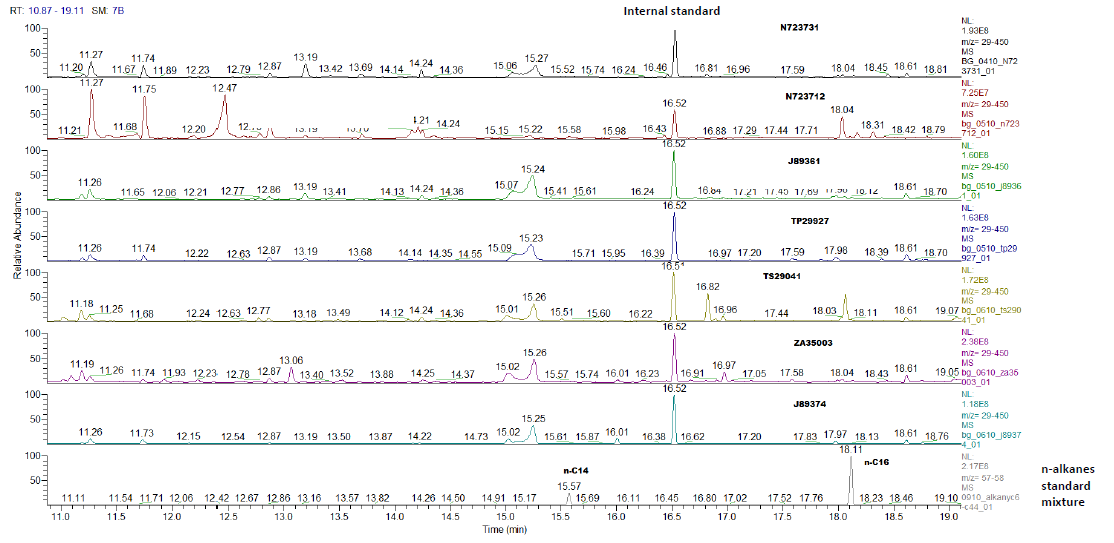
**C)**


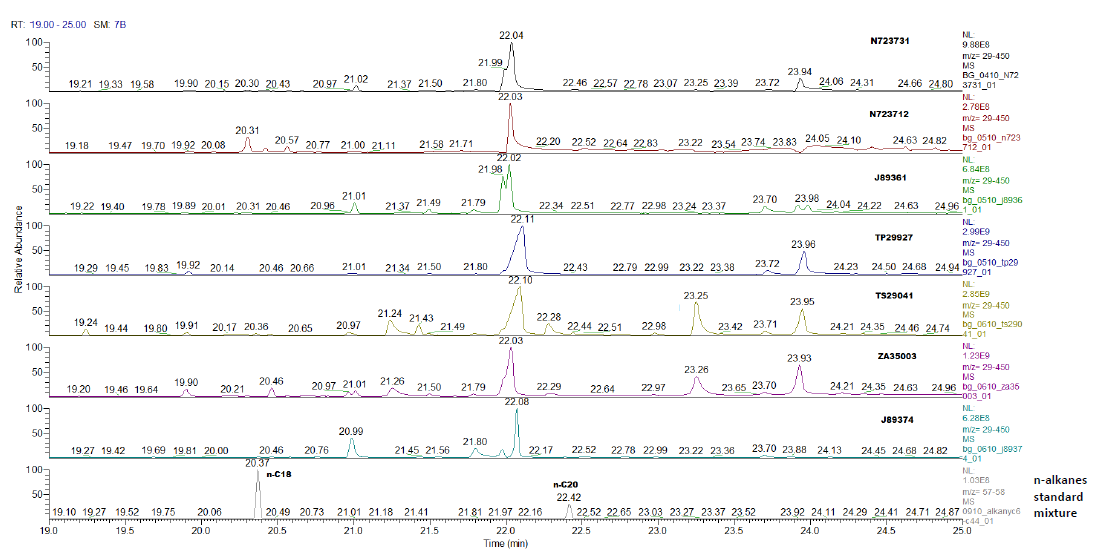
**D)**


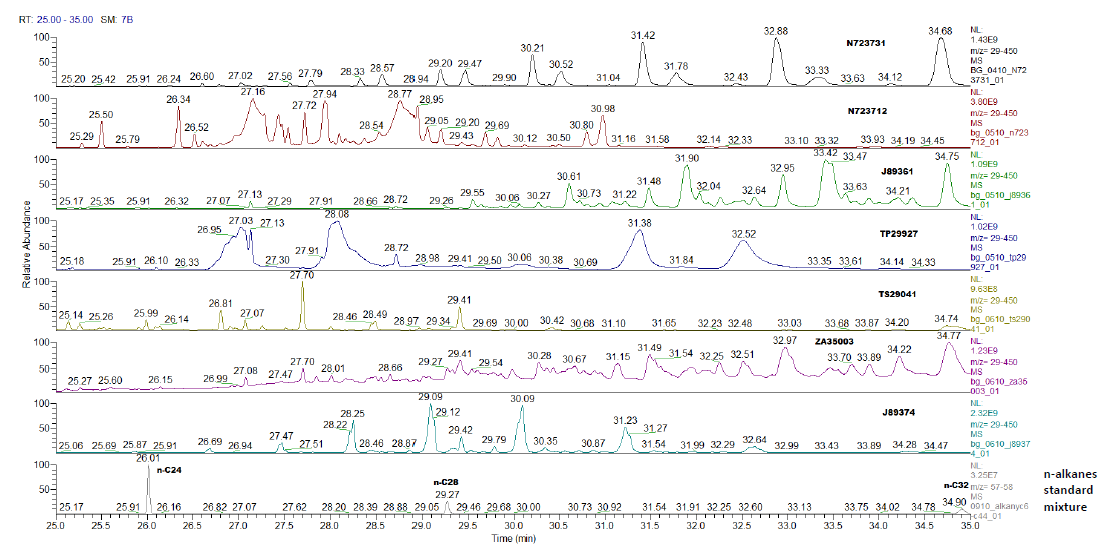
**E)**

**F)**


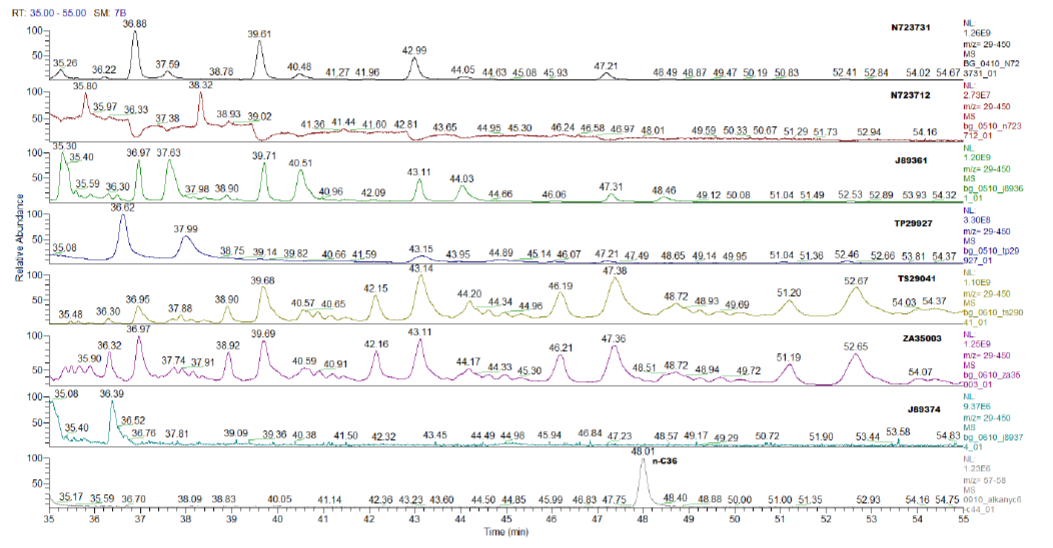


**Fig. S2** Raw GC-MS chromatograms of untargeted preen oil chemical profiling showing chromatograms for: A) complete GC-MS chromatogram for retention times (RTs) of 2.5 - 55 min, and more detailed chromatograms for particular range of RTs: B) RTs = 2.5 - 11 min, C) RTs = 10.87 - 19.11 min with internal standard peak denoted, D) RTs = 19.00 - 25.00 min, E) 25.00 - 35.00 min and F) 35.00 – 55.00 min. Each chromatogram panel represents individual species (Sample ID is written in bold on right) except of bottom panel where retention times and peaks of n-alkanes standard mixture are shown.

**Table S3.** List of (semi-)volatile organic compounds ((s)VOCs) identified in preen oil of eight studied passerine species, including their retention times (RT in min), retention indices (RI), vapour pressure (kPa) at room temperature, and the total number of distinct VOCs for each species (X means presence, empty cell means absence).

| Classification | Compound | RT (min) | RI | *Vapour pressure (kPa)* | *Passer domesticus* | *Sitta europaea* | *Aegithalos caudatus* | *Phoenicurus phoenicurus* | *Locustella luscinioides* | *Acrocephalus arundinaceus* | *Phylloscopus trochilus* |
| --- | --- | --- | --- | --- | --- | --- | --- | --- | --- | --- | --- |
| Alcohol | Pentadecan-1-ol | 20.17 | 1778 | 5.07E-06 |  |  |  |  | X |  |  |
|  | Hexadecan-1-ol | 21.24 | 1880 | 4.08E-07 |  |  |  |  | X | X |  |
|  | Heptadecan-1-ol | 22.28 | 1980 | 3.48E-07 |  |  |  |  | X | X |  |
|  | Octadecan-1-ol | 23.25 | 2082 | 3.60E-07 |  |  |  |  | X | X |  |
|  | Nonadecan-1-ol | 24.21 | 2176 | 4.00E-07 |  |  |  |  | X | X |  |
| Saturated fatty aldehyde | Octanal | 9.53 | 1003 | 1.57E-01 | X |  |  |  |  | X | X |
|  | Nonanal | 11.25 | 1103 | 4.93E-02 | X | X | X | X | X | X | X |
|  | Decanal | 12.87 | 1203 | 1.33E-02 | X | X | X | X | X | X | X |
| Ketone | Heptan-2-one | 7.3 | 870 | 5.13E-01 |  |  |  |  | X |  |  |
|  | Nonan-2-one | 11.02 | 1075 | 8.32E-02 |  |  |  |  | X | X |  |
|  | Tridecan-2-one | 16.82 | 1493 | ? |  |  |  |  | X |  |  |
|  | Tetradecan-2-one | 18.07 | 1597 | 9.33E-04 |  |  |  |  | X |  |  |
|  | Pentadecan-2-one | 19.24 | 1698 | 4.00E-04 |  |  |  |  | X | X |  |
|  | Hexadecan-2-one | 20.36 | 1799 | 4.83E-05 |  |  |  |  | X |  |  |
|  | Heptadecan-2-one | 21.43 | 1899 | 1.33E-04 |  |  |  |  | X | X |  |
|  | Octadecan-2-one | 22.44 | 2004 | 3.83E-05 |  |  |  |  | X | X |  |
| Carboxylic acid | Nonanoic acid | 13.69 | 1275 | 2.20E-04 | X | X |  | X |  |  |  |
|  | Dodecanoic acid | 17.58 | 1555 | 2.13E-06 |  |  |  | X |  | X |  |
|  | Tetradecanoic acid | 19.9 | 1768 | 1.87E-07 | X |  | X | X | X | X |  |
|  | Pentadecanoic acid | 20.97 | 1868 | 8.80E-06 | X |  | X | X | X | X | X |
|  | Hexadecanoic acid | 22.02-22.12 | 1968 | 5.07E-08 | X | X | X | X | X | X | X |
|  | Heptadecanoic acid | 22.98 | 2039 | 4.00E-05 | X |  | X | X | X | X | X |
|  | Octadecanoic acid | 23.89-23.96 | 2155 | 5.33E-09 | X |  | X | X | X | X | X |
| Monoterpene and monoterpene derivatives | Beta-myrcene | 9.24 | 985 | 2.68E-01 |  |  |  |  | X |  |  |
|  | Linalool | 11.2 | 1098 | 2.13E-02 | X | X | X | X | X | X | X |
|  | Geranylacetone | 16.23 | 1450 | 3.35E-03 |  |  |  |  | X |  |  |
| Monounsaturated fatty acid | Tetradec-2-enoic acid | 19.69 | 1730 | 1.69E-06 |  |  |  |  |  | X |  |
|  | Hexadecenoic acid | 21.8 | 1950 | 8.92E-06 | X |  | X | X | X | X | X |
|  | Oleic acid | 23.7 | 2141 | 7.33E-08 | X |  | X | X | X | X | X |
| Ester | Tributyl phosphate | 18.61 | 1655 | 1.51E-04 | X |  | X | X | X | X | X |
|  | Ethyl oleate | 23.98 | 2157 | 4.00E-06 |  |  | X |  |  |  |  |
|  | Dodecyl benzoate | 24.35 | 2182 | ? |  |  |  |  | X | X |  |
|  | Octyl tetradecanoate | 25 | 2290 | ? |  |  |  |  |  |  | X |
|  | Alkyl esters of higher organic acids | 25-55 |  | - |  |  |  |  |  |  |  |
| Phenol | 2,4-ditert-butylphenol | 16.97 | 1503 | 1.00E-03 |  |  |  |  | X | X |  |
| Other | 2,3-dimethylbenzaldehyde | 13.06 | 1210 | 1.27E-02 |  |  |  |  |  | X |  |
|  | 2-Ethylhexyl thioglycolate | 16.01 | 1432 | 2.00E+00 |  |  |  |  |  | X | X |
|  | 2-ethylhexylsalicylate | 20.46 | 1810 | 1.08E-05 |  |  |  |  | X | X |  |
| **Total unique VOCs** |  |  |  |  | 13 | 5 | 12 | 13 | 29 | 27 | 13 |


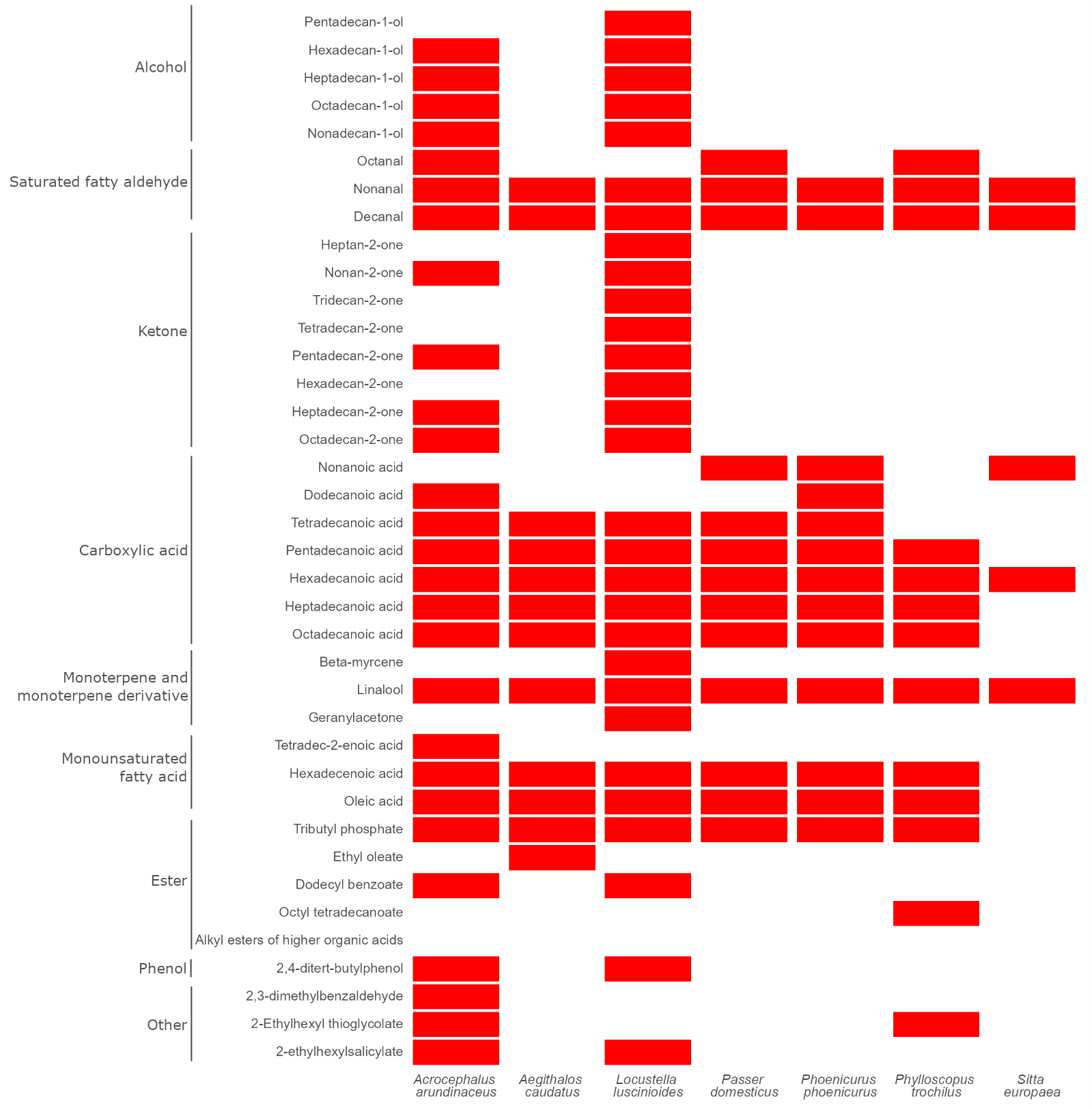


**Fig. S3** Heatmap of (semi-)volatile organic compounds ((s)VOCs) and their higher chemical classification (left row) in preen oil of studied passerine species (red colour means presence, white colour absence of given VOC)


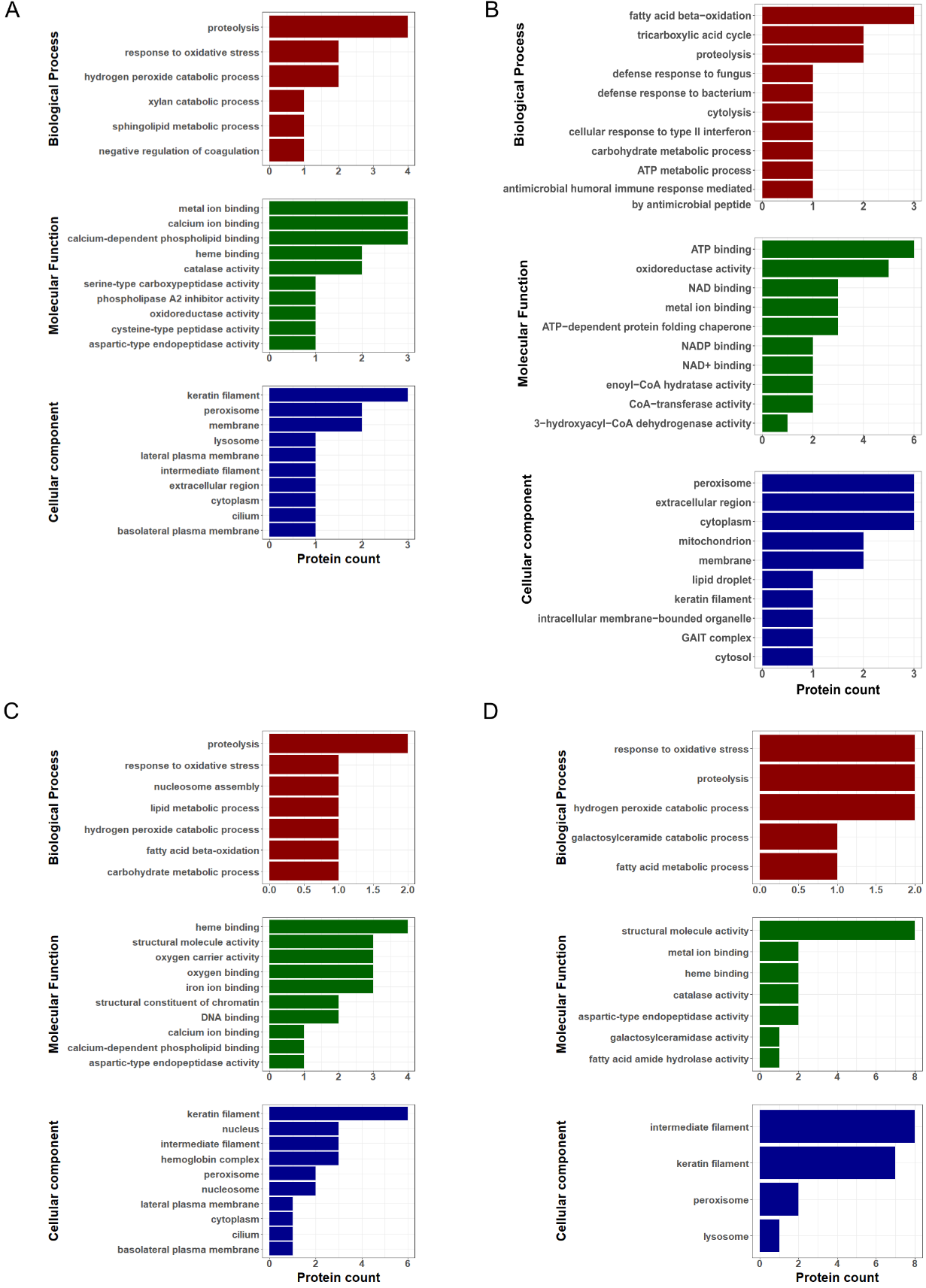


**Fig. S4** Gene ontology (GO) inferred functional properties of proteins detected in preen oil of studied passerine species. A) *Acrocephalus arundinaceus*, B) *Aegithalos caudatus*, C) *Phylloscopus trochilus*, D) *Riparia riparia*


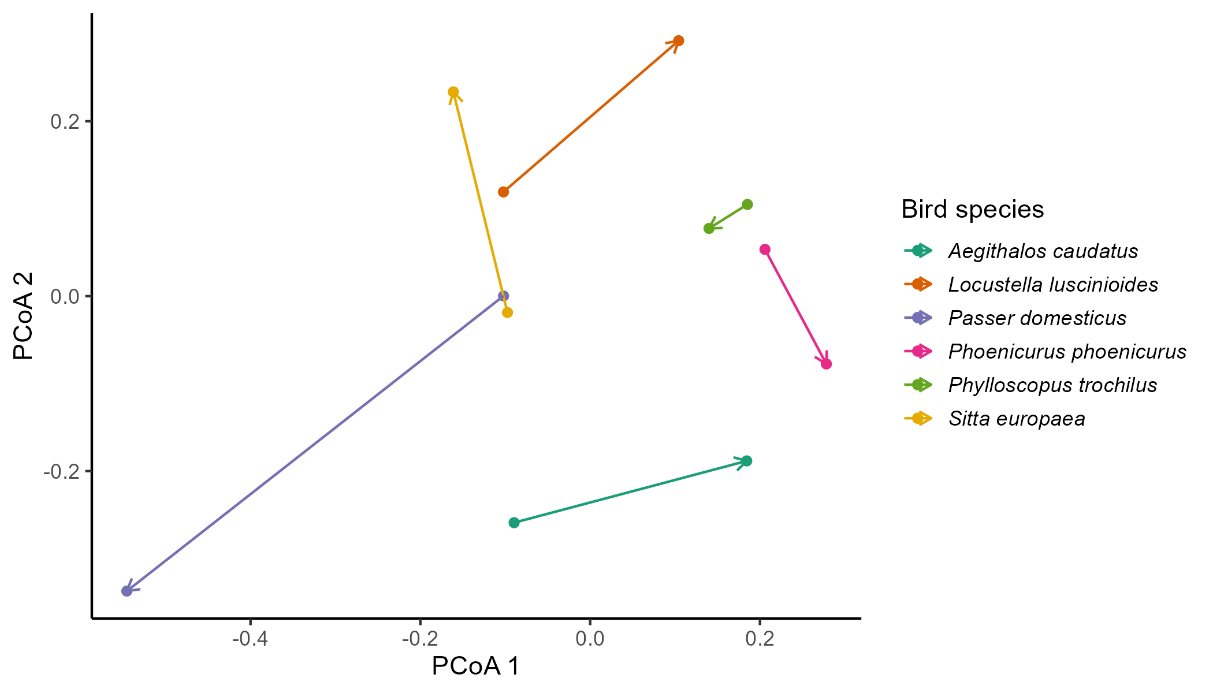

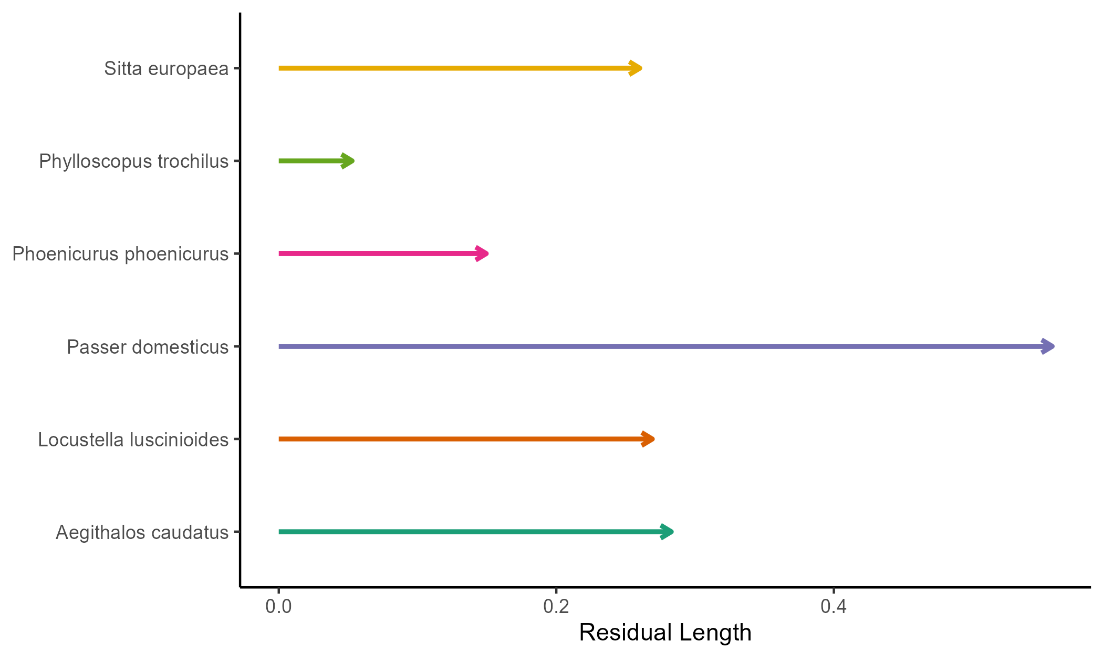


**Fig. S5** Procrustean analysis of preen oil bacteriome principal coordinate analysis (PCoA) (Jaccard). The arrows connect the points from the preen oil bacteriome PCoA (start arrow) to the preen oil VOC profile PCoA (end arrow) (left). The residual lengths for each bird species (right).
